# Global burden of lung cancer attributable to occupational asbestos exposure: 1990 to 2021

**DOI:** 10.1186/s12940-025-01217-z

**Published:** 2025-10-30

**Authors:** Qiulin Huang, Yongran Cheng, Ruijiao Lei, Zijian Chen, Wei Gu, Kari Hemminki, Tianhui Chen

**Affiliations:** 1 Department of Cancer Prevention, Zhejiang Cancer Hospital, Hangzhou, 310022 China; 2 Hangzhou Institute of Medicine (HIM), Chinese Academy of Sciences, Hangzhou, 310018 China; 3 School of Public Health, Hangzhou Normal University, Hangzhou, 311121 China; 4 School of Public Health, Hangzhou Medical College, Hangzhou, 311300 China; 5 Postgraduate training base Alliance of Wenzhou Medical University (Zhejiang Cancer Hospital), Wenzhou, 325000 China; 6 Biomedical Center, Faculty of Medicine in Pilsen, Charles University, Pilsen, Czech Republic; 7 Division of Cancer Epidemiology, German Cancer Research Center (DKFZ), Heidelberg, Germany

**Keywords:** Lung cancer, Global Burden of Disease (GBD) 2021, Occupational asbestos exposure, Chrysotile, Asbestos ban

## Abstract

**Background:**

Asbestos is a well-established occupational carcinogen, with strong evidence linking its exposure to lung cancer. Despite increasing awareness of its health risks, asbestos continues to be used in many countries. We aimed to evaluate the global burden of lung cancer attributable to occupational asbestos exposure and to analyze its epidemiological patterns across time and by regions, sex, and age.

**Methods:**

We utilized lung cancer data from the Global Burden of Disease (GBD) 2021 database, including information on new cases, deaths, and disability-adjusted life-years (DALYs), along with their age-standardized rates by gender and age groups. Temporal trends were examined using Joinpoint regression models with 95% confidence intervals (CIs). The timeline data on global asbestos bans were retrieved from the International Ban Asbestos Secretariat.

**Results:**

We observed, approximately 25 years after the complete ban on asbestos use, a declining trend for lung cancer incidence, as well as for mortality and DALYs due to asbestos exposure. In 2021, occupational asbestos exposure accounted for 9.4% of global lung cancer deaths and 7.2% of DALYs. Between 1990 and 2021, the number of asbestos-related lung cancer deaths increased from 0.13 million to 0.19 million, while DALYs rose from 2.58 million to 3.34 million. The highest deaths and DALYs were observed in regions with high Socio-demographic Index (SDI), though the most rapid increases occurred in lower SDI regions. Over time, lung cancer burden shifted towards older populations, especially those aged over 70.

**Conclusions:**

We found, for the first time, that a complete ban on asbestos with a lag time of 25 years could effectively reduce lung cancer incidence along with asbestos-related deaths and DALYs. These findings underscore the urgent need for a complete ban on asbestos (especially chrysotile).

**Supplementary Information:**

The online version contains supplementary material available at 10.1186/s12940-025-01217-z.

## Introduction

Lung cancer remains the most prevalent cancer globally and the most common cause of death from cancer [1]. Despite advances in medical research and treatment, the overall 5-year relative survival rate for lung cancer has shown minimal improvement in decades, making it one of the cancers with the lowest survival [2, 3]. In 2022, there were 2,480,301 new lung cancer cases and 1,817,172 deaths worldwide [4]. According to 2021 statistics, lung cancer ranked as the seventeenth cause of disability-adjusted life-years (DALYs), a position it has maintained since 2010 [5]. The International Agency for Research on Cancer (IARC) has identified 32 agents with sufficient evidence and 19 agents with limited evidence of carcinogenicity for lung cancer in humans [6]. Specifically, these carcinogens include cigarette smoking; environmental exposures such as environmental tobacco smoke, air pollution, and radon; and occupational carcinogens such as asbestos, crystalline silica, diesel exhaust, polycyclic aromatic hydrocarbons, nickel, chromium, and other metals. Findings from the SYNERGY project have demonstrated that occupational factors and smoking can act synergistically in the causation of lung cancer [7, 8]. Specifically, previous studies have shown that the interaction effects of asbestos exposure and tobacco smoke conform to a multiplicative model for lung cancer risk, which is typical for exposure to almost all types of industrial dusts, especially dust containing crystalline silica [9, 10].

Asbestos, a collective term for a group of fibrous silicates, possesses properties such as heat resistance, acid and alkali resistance, and sound insulation, which led to its extensive use in products such as friction products, thermal insulation, electrical wiring, and building materials [11]. However, asbestos, including all six types (chrysotile, crocidolite, amosite, tremolite, actinolite, and anthophyllite), has been classified as a Group 1 carcinogen by IARC, with sufficient evidence in humans for causing lung cancer, mesothelioma, and other cancers [12, 13]. The Link between asbestos and lung cancer was first discovered in 1930 [14], and afterwards many studies have confirmed [15–18]. In countries with historically high asbestos use, especially crocidolite, amosite and anthophyllite (amphibole group of asbestos), lung cancer attributable to occupational asbestos exposure has been estimated to account for approximately 3–8% of all lung cancer cases [19].

Although some developed countries have implemented a complete ban on asbestos use, BRIC countries (Brazil, Russia, India, and China) are still producing and using large amounts of chrysotile asbestos [20–23]. Therefore, this study aims to investigate the burden of lung cancer and its association with occupational exposure to asbestos using the most recent data from the Global Burden of Disease (GBD) 2021 database. These findings may provide valuable insights into the association of asbestos exposure with lung cancer burden and underscore the urgent need for a complete ban on asbestos (especially chrysotile).

## Materials and methods

We extracted the lung cancer related number of new cases, deaths, DALYs and their corresponding age-standardized rates (ASRs) during 1990–2021 from the GBD 2021 online results tool [24], which was engineered by the Institute for Health Metrics and Evaluation. The previous studies have reported the general methods of the GBD study [25, 26]. A detailed description of the methodology for estimating lung cancer burden is provided in the supplementary material (Supplementary Text 1). Patients were divided into three age groups: 20–49 years, 50–69 years, and over 69 years. Individuals younger than 20 years were excluded due to the unavailability of data for this age group.

We used the timeline data on global asbestos bans and restrictions provided by the International Ban Asbestos Secretariat to obtain a list of countries that have prohibited the use of asbestos [27]. After excluding countries not covered by the GBD data and those with unclear ban dates, we ultimately included 50 countries with a complete asbestos ban (Supplementary Table 1). The United States of America (USA) was also excluded because the regulation banning the use of chrysotile asbestos was adopted last year but has not yet taken effect. We descriptively examined the time trends in incident new cases of lung cancer along with asbestos-induced lung cancer deaths and DALYs following the implementation of the complete ban on asbestos use.

We used the Joinpoint Regression Program (version 5.1.0.0) to quantify time trends in a structured manner and test which trends between joinpoints are statistically significant [28]. The overall trends in lung cancer burden attributable to occupational asbestos exposure were reflected by the annual percentage change (APC), average APC (AAPC), and their respective 95% confidence intervals (CIs) between successive joinpoints [29].

Age-standardized rates (ASRs) were calculated by summing the products of age-specific ratios and standard population proportions, allowing for the comparison of mortality and DALY rates across nations with different age compositions. To capture the temporal trend of age-standardized rates (ASRs), we calculated the estimated annual percentage changes (EAPC) values and their corresponding 95% CI for the period from 1990 to 2021 [30]. First, we established a linear regression model defined as y = α + βx + ε, where y represents the natural logarithm of the ASR, x denotes the calendar year, and ε is the error term. The EAPC was then calculated using the formula 100 × (exp (β)—1). If the estimated EAPC value and its lower 95% CI were greater than 0, the ASRs were considered to be increasing. Conversely, if both the EAPC and its upper 95% CI were less than 0, a declining trend was considered. In cases where neither condition was met, the ASR was considered stable over the analysis period.

Spearman correlation analysis was conducted to assess the relationships between EAPC, ASRs, and Socio-demographic Index (SDI) levels. Additionally, a Locally Estimated Scatterplot Smoothing (LOESS) smoother was applied to visualize these associations. We specifically examined the correlations of EAPC with ASR in 1990 and SDI in 2021 to evaluate the impact of baseline burden and the comprehensive development level of countries/regions on the trends in lung cancer burden attributable to occupational asbestos exposure.

All statistical analyses were performed using the R (version 4.3.1) and a two-tailed *P* < 0.05 was considered statistically significant.

## Results

### Global, regional, and national trends in lung cancer burden attributable to occupational asbestos exposure

**Fig. 1:**
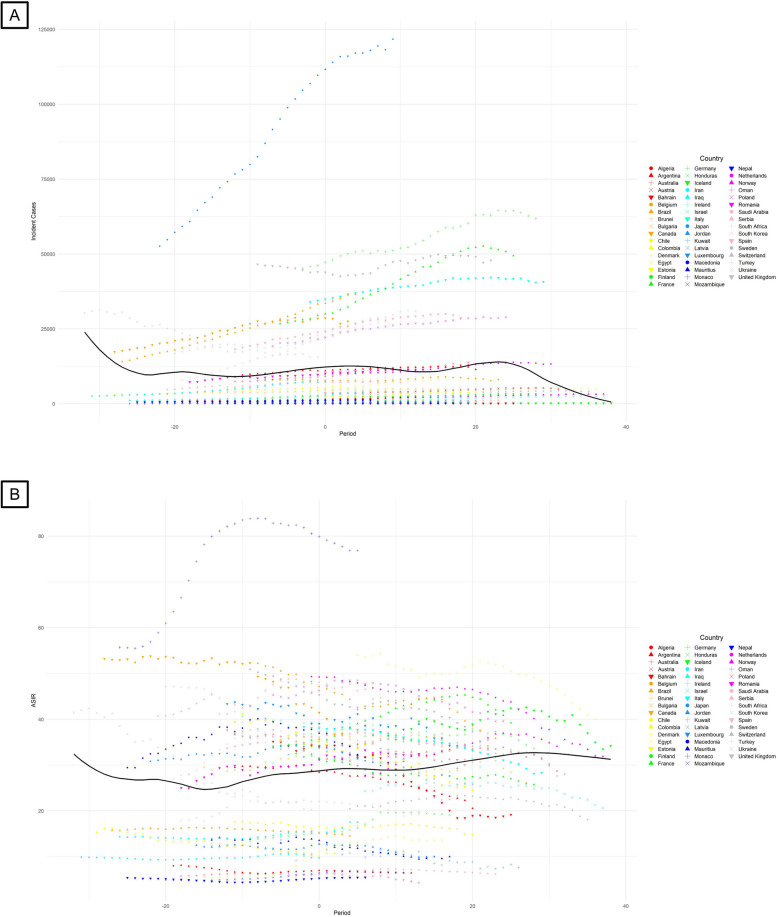
Temporal trends in lung cancer new cases (**A**) and ASIR (**B**) with complete asbestos bans. ASIR, age-standardized incidence rate. The solid black curve reflects the overall trend of incident cases and ASIR

**Fig. 2:**
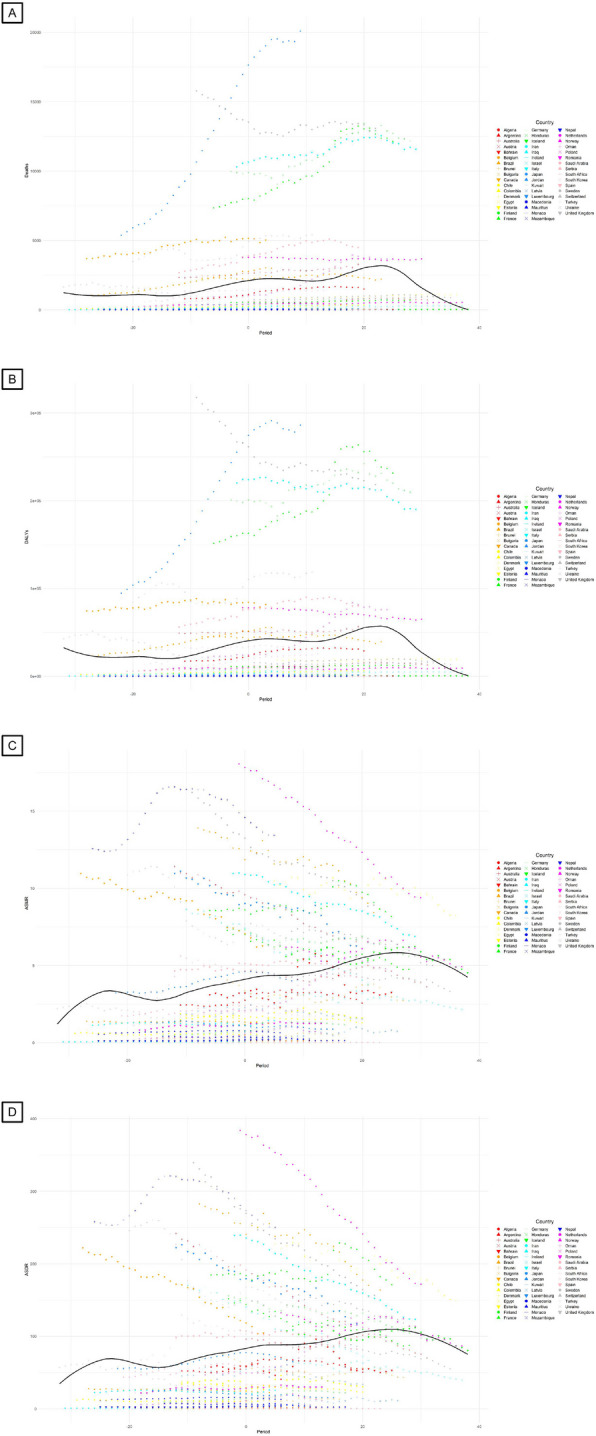
Temporal trends in lung cancer burden attributable to occupational asbestos exposure with complete asbestos bans. **A** Lung cancer deaths; **B** Lung cancer DALYs; **C** Lung cancer ASMR; **D** Lung cancer ASDR. DALYs, disability-adjusted life-years; ASMR, age-standardized mortality rate; ASDR, age-standardized rate of disability-adjusted life-years. The solid black curve reflects the overall trend of deaths and DALYs and their corresponding age-standardized rates

We found, for the first time, that the number of incident new cases and age-standardized incidence rate (ASIR) started to decline after 25 years’ complete ban on asbestos use (Fig. 1). We also found that the lung cancer deaths (Fig. 2A), DALYs (Fig. 2B), age-standardized mortality rate (ASMR; Fig. 2C) and age-standardized DALYs rate (ASDR; Fig. 2D) due to occupational asbestos exposure decline starting from 25 years’ complete ban on asbestos use. Iceland was the first country to implement a comprehensive ban on asbestos. Despite this, the incidence of lung cancer continued to rise from 1990, with the ASIR remaining stable. However, 27 years after the ban, Iceland experienced a notable decline in both new cases and the ASIR. In countries with high burden of asbestos-related lung cancer, such as Germany, France, and Italy, the upward trend in lung cancer incidence, as well as deaths and DALYs attributed to asbestos, plateaued around 20 years after a complete ban on asbestos.

**Fig. 3:**
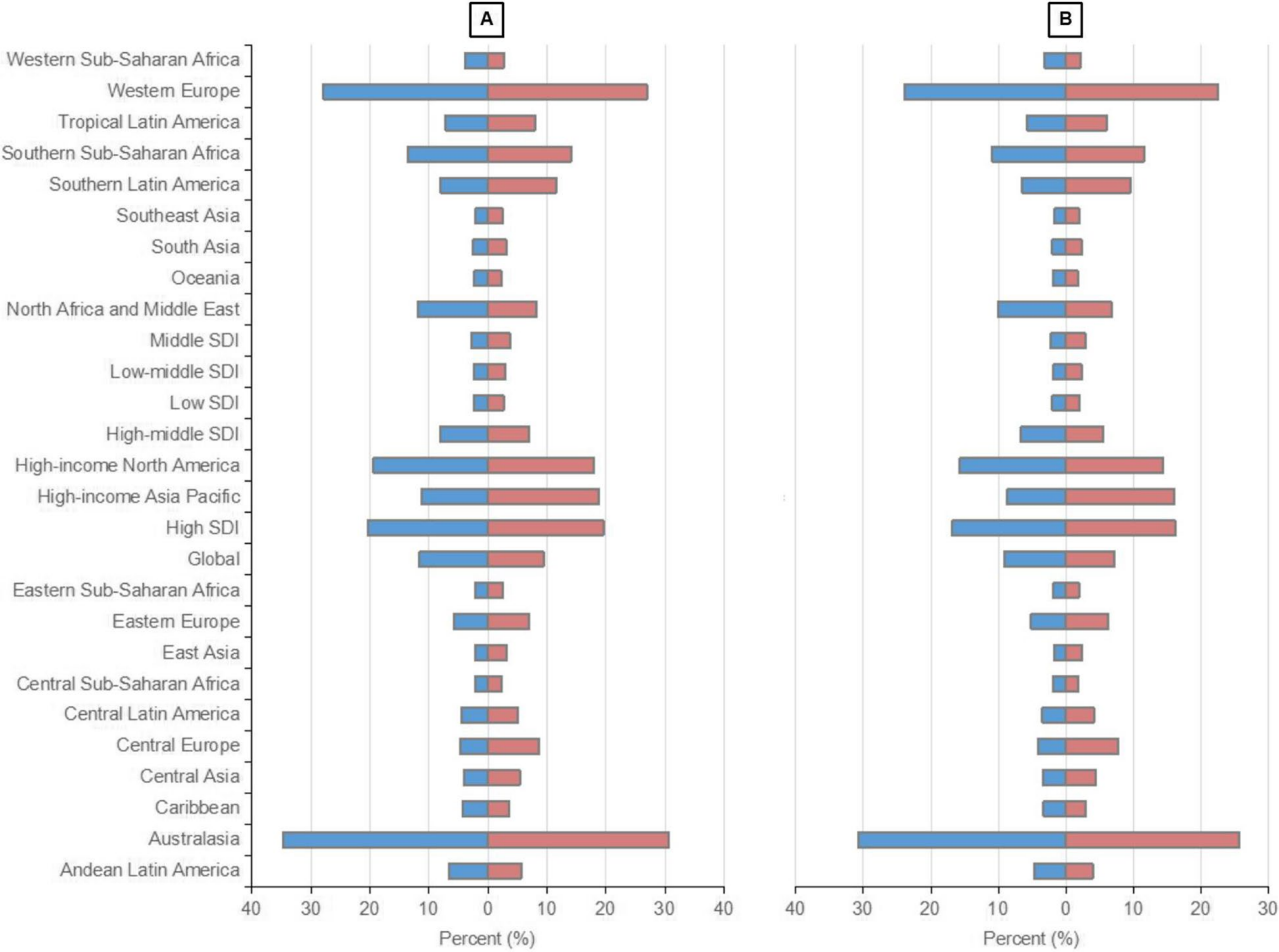
Proportion of asbestos-related lung cancer deaths (**A**) and DALYs (**B**) in 1990 and 2021. DALYs, disability-adjusted life-years; GBD, Global Burden of Disease

In 2021, lung cancer caused a total of 2,016,547 deaths and 46,536,272 DALYs globally, compared to 1,080,128 deaths and 28,459,836 DALYs in 1990. Occupational asbestos exposure accounted for 9.4% of lung cancer deaths and 7.2% of lung cancer DALYs in 2021 (Fig. 3). From 1990 to 2021, the number of lung cancer deaths attributed to occupational asbestos exposure increased from 0.13 million to 0.19 million while DALYs rose from 2.58 million to 3.34 million. Conversely, the ASMR and ASDR in 2021 were 2.28 and 39.07, respectively, indicating a significant decrease compared to 1990, with EAPC of −1.17 and −1.63, respectively (Table 1 and Supplementary Table 2). This divergence between absolute numbers and ASRs primarily reflects the combined effect of population growth and aging.

**Table 1:** Deaths and age-standardized mortality rates of asbestos-related lung cancer from 1990 to 2021

Variable	No. (95% UI)				No. (95% CI)
	**1990**		**2021**		**1990–2021**
	**Death number, × 10**^**3**^	**ASMR per 100,000**	**Death number, × 10**^**3**^	**ASMR per 100,000**	**EAPC of ASMR**
Global	125.16(89.38 to 161.89)	3.42(2.45 to 4.40)	189.40(132.32 to 244.50)	2.28(1.57 to 2.94)	−1.17(−1.29 to −1.04)
SDI					
Low	0.33(0.11 to 0.68)	0.17(0.06 to 0.34)	0.76(0.29 to 1.47)	0.18(0.07 to 0.34)	0.26(0.1 to 0.42)
Low-middle	1.23(0.71 to 1.89)	0.24(0.14 to 0.36)	4.07(2.49 to 6.01)	0.32(0.20 to 0.47)	1.10(1.05 to 1.14)
Middle	6.38(4.00 to 9.35)	0.77(0.49 to 1.11)	22.50(14.28 to 32.92)	0.94(0.58 to 1.38)	0.86(0.63 to 1.1)
High-middle	28.99(19.38 to 38.80)	2.98(2.01 to 3.97)	45.24(30.93 to 61.04)	2.27(1.55 to 3.06)	−0.75(−0.92 to −0.59)
High	88.12(64.01 to 112.51)	7.60(5.51 to 9.72)	116.65(83.01 to 147.70)	4.95(3.57 to 6.30)	−1.26(−1.38 to −1.15)
Regional					
Andean Latin America	0.18(0.11 to 0.28)	1.03(0.64 to 1.54)	0.36(0.20 to 0.56)	0.64(0.36 to 1.00)	−1.96(−2.36 to −1.55)
Australasia	2.75(2.07 to 3.35)	11.19(8.44 to 13.69)	3.76(2.78 to 4.63)	6.35(4.67 to 7.85)	−1.88(−1.98 to −1.78)
Caribbean	0.24(0.15 to 0.35)	0.99(0.62 to 1.44)	0.39(0.23 to 0.60)	0.72(0.42 to 1.11)	−0.98(−1.13 to −0.84)
Central Asia	0.54(0.33 to 0.78)	1.17(0.71 to 1.68)	0.59(0.36 to 0.88)	0.80(0.49 to 1.17)	−1.27(−1.42 to −1.13)
Central Europe	2.81(1.73 to 4.03)	1.81(1.11 to 2.59)	7.03(4.56 to 9.84)	2.95(1.91 to 4.15)	2.32(2.06 to 2.59)
Central Latin America	0.53(0.34 to 0.74)	0.73(0.47 to 1.02)	1.25(0.80 to 1.81)	0.52(0.34 to 0.76)	−1.01(−1.18 to −0.85)
Central Sub-Saharan Africa	0.05(0.01 to 0.14)	0.27(0.05 to 0.71)	0.12(0.02 to 0.33)	0.27(0.05 to 0.75)	0.01(−0.38 to 0.41)
East Asia	6.34(3.78 to 9.70)	0.96(0.58 to 1.44)	26.66(16.17 to 40.13)	1.34(0.81 to 2.01)	1.69(1.29 to 2.09)
Eastern Europe	5.80(3.60 to 8.24)	1.99(1.23 to 2.81)	5.26(3.23 to 7.78)	1.44(0.88 to 2.12)	−1.44(−1.66 to −1.23)
Eastern Sub-Saharan Africa	0.12(0.02 to 0.32)	0.18(0.03 to 0.47)	0.26(0.05 to 0.65)	0.20(0.04 to 0.48)	0.23(0.07 to 0.39)
High-income Asia Pacific	5.77(3.84 to 7.83)	3.03(2.02 to 4.10)	21.90(14.51 to 29.24)	3.63(2.41 to 4.84)	1.03(0.77 to 1.29)
High-income North America	33.65(23.94 to 43.27)	8.90(6.31 to 11.49)	35.59(25.21 to 45.22)	4.98(3.52 to 6.32)	−2.15(−2.41 to −1.89)
North Africa and Middle East	3.72(2.22 to 5.72)	2.45(1.49 to 3.70)	6.19(3.61 to 9.71)	1.58(0.92 to 2.45)	−1.45(−1.8 to −1.11)
Oceania	0.01(0.01 to 0.02)	0.52(0.26 to 0.87)	0.03(0.01 to 0.05)	0.53(0.25 to 0.91)	0.20(0.08 to 0.33)
South Asia	0.88(0.46 to 1.43)	0.18(0.10 to 0.29)	3.12(1.69 to 4.95)	0.23(0.12 to 0.36)	0.61(0.45 to 0.76)
Southeast Asia	1.02(0.58 to 1.60)	0.50(0.28 to 0.77)	3.35(1.95 to 5.24)	0.62(0.36 to 0.98)	0.35(0.21 to 0.48)
Southern Latin America	1.09(0.67 to 1.54)	2.37(1.46 to 3.34)	2.01(1.32 to 2.76)	2.22(1.45 to 3.05)	0.44(0.14 to 0.74)
Southern Sub-Saharan Africa	0.63(0.40 to 0.91)	2.55(1.64 to 3.63)	1.54(1.01 to 2.09)	2.98(2.00 to 4.01)	0.21(−0.52 to 0.93)
Tropical Latin America	1.06(0.69 to 1.44)	1.36(0.90 to 1.84)	3.07(2.03 to 4.10)	1.25(0.83 to 1.67)	0.02(−0.07 to 0.11)
Western Europe	57.84(42.34 to 73.11)	9.44(6.89 to 11.97)	66.73(48.62 to 82.76)	6.42(4.69 to 8.02)	−0.99(−1.11 to −0.87)
Western Sub-Saharan Africa	0.12(0.05 to 0.20)	0.14(0.06 to 0.24)	0.21(0.10 to 0.37)	0.13(0.06 to 0.22)	−0.51(−0.68 to −0.35)

*UI* Uncertainty interval, *CI* Confidence interval, *ASMR* Age‐standardized mortality rate, *EAPC* Estimated annual percentage change

Lung cancer deaths and DALYs caused by occupational asbestos exposure across different SDI regions shows great disparities. In 2021, High SDI regions had the highest number of lung cancer deaths and DALYs attributable to occupational asbestos exposure, accounting for 62.6% and 58.5% of the global total, respectively. High-middle SDI regions followed, with deaths and DALYs approximately half of that of High SDI regions (Supplementary Fig. 1). The ASMR and ASDR in 2021 also exhibited a similar distribution (Supplementary Fig. 2). However, when considering temporal trends, while the number of deaths and DALYs increased in all SDI regions, only the ASRs in High and High-middle SDI regions showed a significant decrease, with the decline being more pronounced in High SDI regions (Tables 1 and 2 and Supplementary Tables 2 and 3).

**Table 2:** Joinpoint regression analysis of ASMR in SDI regions from 1990 to 2021

	**Both sexes**			**Male**			**Female**		
	**Period**	**APC/AAPC (95% CI)**	***p*-values**	**Period**	**APC/AAPC (95% CI)**	***p*-values**	**Period**	**APC/AAPC (95% CI)**	***p*-values**
Global	1990 ~ 1995	−0.6 (−0.9 to 0.1)	0.065	1990 ~ 1995	−0.9 (−1.1 to −0.5)	0.0012	1990 ~ 1995	1.1 (0.9 to 1.5)	< 0.0001
	1995 ~ 1998	−1.9 (−2.2 to −0.9)	0.0076	1995 ~ 1998	−2.3 (−2.5 to −1.7)	< 0.0001	1995 ~ 1998	−0.4 (−0.7 to 0.2)	0.19
	1998 ~ 2006	−0.8 (−1.2 to −0.4)	0.0076	1998 ~ 2012	−0.9 (−0.9 to −0.8)	< 0.0001	1998 ~ 2006	0.7 (0.6 to 0.9)	0.0024
	2006 ~ 2012	−0.3 (−3.1 to 0.1)	0.11	2012 ~ 2016	−2.6 (−2.8 to −1.3)	< 0.0001	2006 ~ 2009	2.2 (1.6 to 2.6)	< 0.0001
	2012 ~ 2021	−2.7 (−3.1 to −2.4)	< 0.0001	2016 ~ 2019	−3.8 (−4.2 to −3.3)	< 0.0001	2009 ~ 2013	0.4 (−0.3 to 0.8)	0.16
				2019 ~ 2021	−1.5 (−2.3 to −0.9)	< 0.0001	2013 ~ 2021	−1.5 (−1.7 to −1.4)	< 0.0001
	Full Range	−1.3 (−1.4 to −1.3)	< 0.0001	Full Range	−1.6 (−1.6 to −1.5)	< 0.0001	Full Range	0.2 (0.2 to 0.2)	< 0.0001
High SDI	1990 ~ 2013	−0.9 (−1.0 to −0.9)	< 0.0001	1990 ~ 2013	−1.5 (−1.5 to −1.4)	< 0.0001	1990 ~ 1995	1.7 (1.3 to 2.6)	0.00080
	2013 ~ 2021	−2.8 (−3.1 to −2.5)	< 0.0001	2013 ~ 2021	−3.2 (−3.5 to −2.9)	< 0.0001	1995 ~ 1998	−0.1 (−0.6 to 0.9)	0.92
							1998 ~ 2013	1.1 (0.9 to 1.4)	0.037
							2013 ~ 2021	−1.8 (−2.1 to −1.4)	0.0016
	Full Range	−1.4 (−1.5 to −1.4)	< 0.0001	Full Range	−1.9 (−2.0 to −1.9)	< 0.0001	Full Range	0.3 (0.3 to 0.4)	< 0.0001
High-middle SDI	1990 ~ 1994	1.4 (0.8 to 2.3)	0.0020	1990 ~ 1994	1.1 (0.5 to 1.8)	< 0.0001	1990 ~ 1994	0.9 (0.5 to 1.7)	0.0040
	1994 ~ 1998	−2.0 (−2.8 to −1.2)	0.012	1994 ~ 1999	−1.9 (−2.8 to −1.4)	< 0.0001	1994 ~ 1998	−1.0 (−1.9 to −0.5)	0.0052
	1998 ~ 2006	−0.3 (−0.9 to 0.3)	0.20	1999 ~ 2012	−0.2 (−0.3 to −0.1)	0.0080	1998 ~ 2007	1.3 (1.1 to 1.6)	0.00040
	2006 ~ 2012	0.4 (−3.3 to 1.1)	0.24	2012 ~ 2021	−3.2 (−3.4 to −3.0)	< 0.0001	2007 ~ 2010	4.6 (3.3 to 5.1)	0.0024
	2012 ~ 2021	−2.8 (−3.3 to −2.1)	< 0.0001				2010 ~ 2021	−0.5 (−0.7 to −0.4)	0.00080
	Full Range	−0.9 (−1.0 to −0.8)	< 0.0001	Full Range	−1.2 (−1.3 to −1.2)	< 0.0001	Full Range	0.6 (0.6 to 0.7)	< 0.0001
Low SDI	1990 ~ 1996	0.2 (−0.1 to 0.4)	0.092	1990 ~ 1996	0.2 (0.0 to 0.4)	0.070	1990 ~ 1995	1.6 (1.0 to 2.7)	0.0032
	1996 ~ 2002	−1.0 (−1.4 to 0.3)	0.073	1996 ~ 2002	−0.8 (−1.3 to −0.6)	0.026	1995 ~ 1999	0.4 (−0.6 to 5.0)	0.26
	2002 ~ 2008	−0.1 (−1.1 to 0.1)	0.18	2002 ~ 2008	0.0 (−0.5 to 0.3)	0.59	1999 ~ 2003	5.1 (0.7 to 6.0)	0.0048
	2008 ~ 2012	1.1 (−0.2 to 1.4)	0.15	2008 ~ 2012	1.3 (0.2 to 1.6)	0.012	2003 ~ 2011	1.0 (0.7 to 1.4)	0.0076
Low SDI	2012 ~ 2016	2.0 (1.0 to 2.3)	0.0024	2012 ~ 2017	2.0 (1.8 to 2.4)	< 0.0001	2011 ~ 2014	6.2 (5.3 to 6.7)	0.0016
	2016 ~ 2019	1.0 (0.8 to 1.9)	< 0.0001	2017 ~ 2021	0.5 (0.2 to 0.8)	0.014	2014 ~ 2018	3.4 (2.7 to 3.9)	< 0.0001
	2019 ~ 2021	−0.3 (−0.8 to 0.3)	0.24				2018 ~ 2021	0.9 (−0.1 to 1.6)	0.073
	Full Range	0.3 (0.3 to 0.3)	< 0.0001	Full Range	0.4 (0.4 to 0.5)	< 0.0001	Full Range	2.3 (2.3 to 2.4)	< 0.0001
Low-middle SDI	1990 ~ 2009	1.2 (1.1 to 1.3)	0.0016	1990 ~ 2019	1.3 (1.2 to 1.3)	< 0.0001	1990 ~ 1999	0.9 (0.7 to 1.0)	0.0024
	2009 ~ 2012	0.4 (0.1 to 1.0)	0.017	2019 ~ 2021	−1.5 (−2.7 to 0.0)	0.047	1999 ~ 2002	3.1 (1.1 to 3.4)	0.0016
	2012 ~ 2019	1.5 (1.3 to 2.1)	< 0.0001				2002 ~ 2010	2.1 (2.0 to 2.4)	< 0.0001
	2019 ~ 2021	−2.0 (−2.9 to −0.9)	0.00080				2010 ~ 2013	0.9 (0.5 to 1.5)	< 0.0001
							2013 ~ 2016	3.7 (3.2 to 4.2)	< 0.0001
							2016 ~ 2019	1.4 (1.0 to 1.9)	< 0.0001
							2019 ~ 2021	−2.1 (−2.8 to −1.4)	< 0.0001
	Full Range	1.0 (0.9 to 1.0)	< 0.0001	Full Range	1.1 (1.0 to 1.1)	< 0.0001	Full Range	1.5 (1.5 to 1.6)	< 0.0001
Middle SDI	1990 ~ 1997	1.8 (1.5 to 2.5)	0.00040	1990 ~ 1997	1.9 (1.0 to 2.8)	0.021	1990 ~ 2007	0.9 (0.8 to 1.0)	< 0.0001
	1997 ~ 2007	1.0 (0.5 to 1.2)	0.016	1997 ~ 2007	1.1 (−0.4 to 2.9)	0.098	2007 ~ 2011	5.2 (4.6 to 6.3)	< 0.0001
	2007 ~ 2011	3.9 (3.3 to 4.9)	0.0028	2007 ~ 2010	4.8 (0.9 to 5.5)	0.017	2011 ~ 2015	−2.4 (−3.3 to −1.8)	< 0.0001
	2011 ~ 2018	−2.3 (−2.8 to −2.1)	0.0032	2010 ~ 2013	−0.8 (−1.7 to 5.1)	0.25	2015 ~ 2021	1.1 (0.6 to 1.7)	< 0.0001
	2018 ~ 2021	0.0 (−1.1 to 1.5)	0.99	2013 ~ 2018	−2.8 (−4.2 to −1.9)	0.014			
				2018 ~ 2021	−0.3 (−1.8 to 1.4)	0.59			
	Full Range	0.7 (0.6 to 0.8)	< 0.0001	Full Range	0.7 (0.6 to 0.8)	< 0.0001	Full Range	1.1 (1.0 to 1.1)	< 0.0001

*ASMR* Age-standardized mortality rate, *SDI* Socio-demographic Index, *APC* Annual percentage change, *AAPC* Average annual percentage change, *CI* Confidence interval

Among the Geographic regions, Australasia had the highest proportion of lung cancer deaths and DALYs attributable to occupational exposure to asbestos, with 30.6% and 25.8%, respectively (Fig. 3). In terms of absolute numbers, Western Europe led by a significant margin in both deaths and DALYs, with an increase in death numbers despite a slight reduction in DALYs from 1990 to 2021 (Supplementary Fig. 3). Although Australasia had relatively fewer deaths and DALYs, its ASRs ranked among the highest globally, comparable to Western Europe. Notably, the EAPCs of both Australasia and Western Europe were significantly less than zero, indicating a marked decline in ASRs—a trend similarly observed in regions such as Andean Latin America, High-income North America, and North Africa and Middle East (Supplementary Fig. 4).

**Fig. 4:**
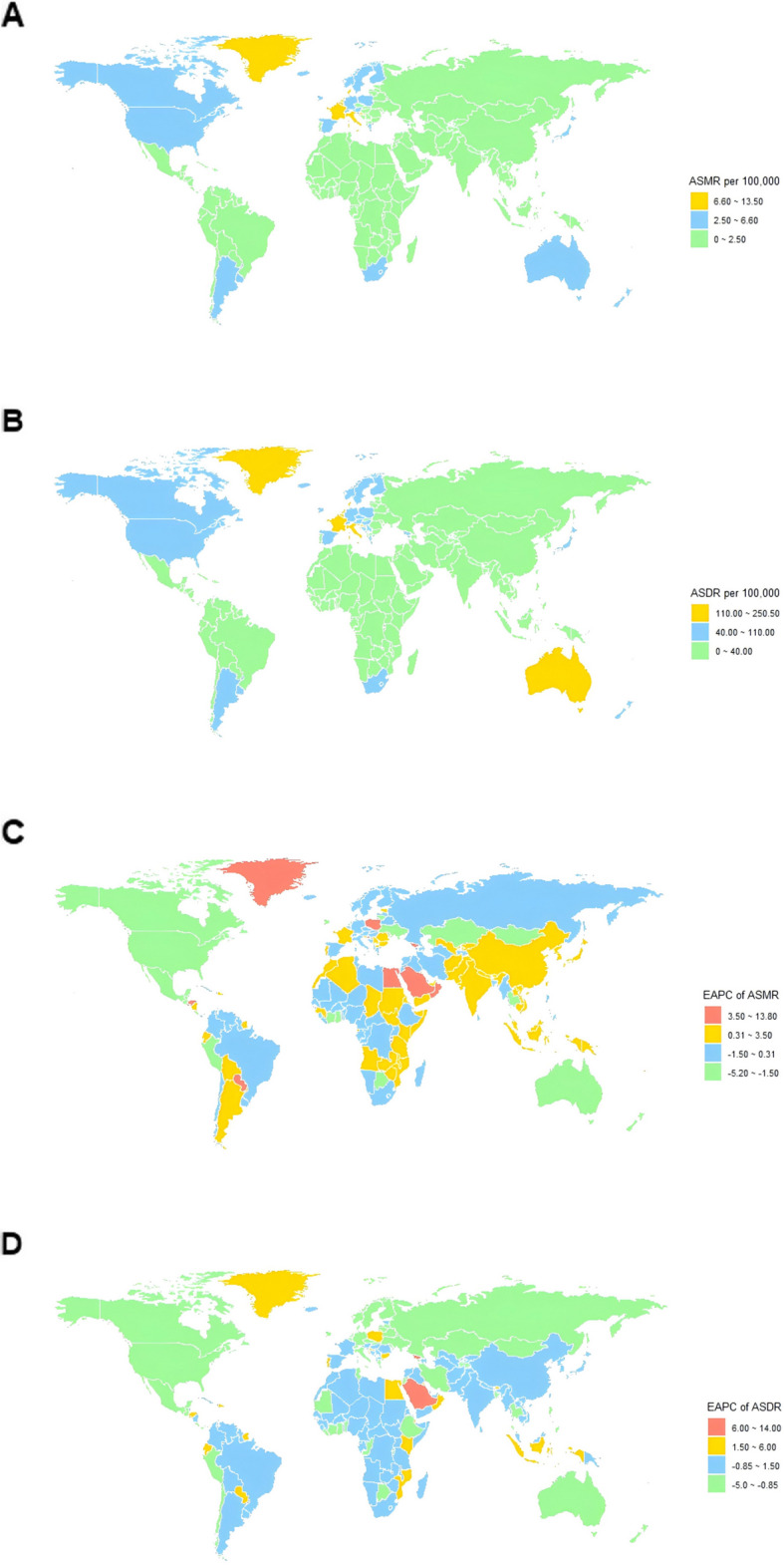
The global epidemiological patterns of lung cancer burden attributable to occupational asbestos exposure. **A** ASMR in 2021; **B** ASDR in 2021; **C** EAPC in ASMR from 1990 to 2021; **D** EAPC in ASDR from 1990 to 2021. ASMR, age-standardized mortality rate; ASDR, age-standardized rate of disability-adjusted life-years; EAPC, estimated annual percentage change

At the national level, the United States, China, and Japan ranked as the top three countries in lung cancer deaths and DALYs due to occupational asbestos exposure, collectively accounting for approximately 40% of the global burden in 2021 (Supplementary Tables 4 and 5). Georgia was the fastest-growing country in both ASMR and ASDR, with EAPCs of 13.72 (95% CI: 11.56 to 15.91) and 13.76 (95% CI: 11.59 to 15.98), respectively (Fig. 4).

### Associations of time trend with baseline ASRs and SDI

Based on Supplementary Fig. 5, there is an overall positive correlation between lung cancer ASMR attributable to occupational asbestos exposure and SDI. From 1990 to 2021, the observed global ASMR consistently exceeded the anticipated values; this trend was also evident in regions such as Western Europe and High-income North America, although their ASMR showed a sharp decline over time. A similar trend was observed in the relationship between ASDR and SDI (Supplementary Fig. 6).

**Fig. 5:**
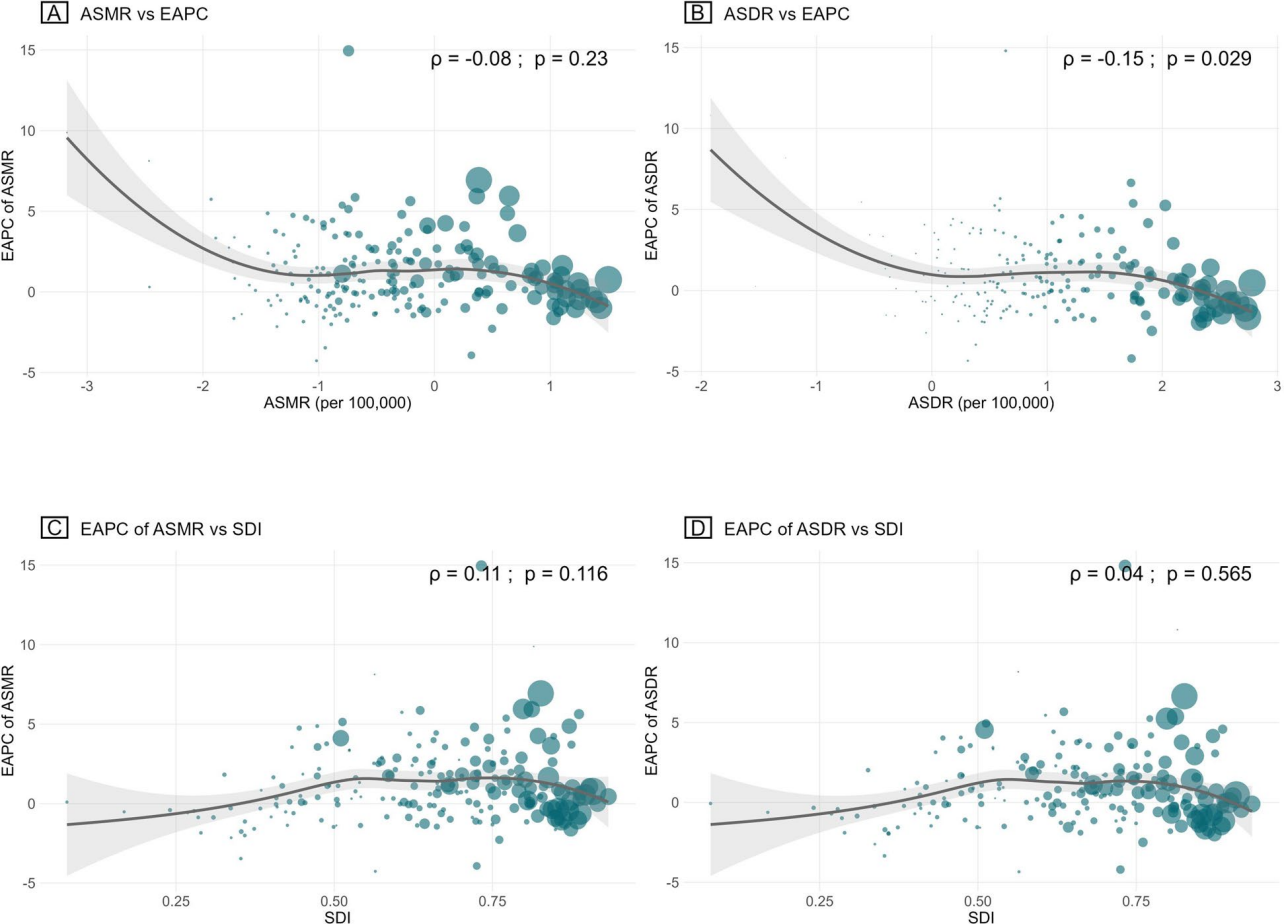
Correlation of EAPC in lung cancer deaths and DALYs attributable to occupational asbestos exposure with age-standardized rates in 1990 and SDI in 2021. The ρ indices and *P* values were derived using Spearman correlation analysis. Larger circles represent a greater number of deaths or DALYs in 2021. EAPC, estimated annual percentage change; DALYs, disability-adjusted life-years; SDI, Socio-demographic Index

The EAPC of both ASMR and ASDR was negatively correlated with baseline ASRs in 1990 across various countries. However, this trend is particularly prominent only when the ASRs were near the extremes. Overall, EAPC demonstrated a slight positive correlation with SDI, although a certain negative correlation emerged when SDI reached a high level (Fig. 5).

### Age and gender-specific burden of lung cancer attributable to occupational asbestos exposure

From 1990 to 2021, lung cancer deaths and DALYs attributable to occupational asbestos exposure were extremely rare among individuals under 50 years old across all regions. In 2021, deaths and DALYs had become more concentrated among older populations compared to thirty years ago (Supplementary Fig. 7). This shift toward older age groups may reflect demographic changes, including population aging and increased life expectancy over time. When analyzing the data by sex, it is evident that the proportion of deaths and DALYs over 70 years old has steadily increased for both male and female from 1990 to 2021. However, the proportion of lung cancer deaths and DALYs in female patients in this age group is larger than that of male (Supplementary Fig. 8).

In 2021, the age-specific mortality rates for both sexes and for males peaked in the 90–94 age group, while for females, the mortality rates increased monotonically with age. The age-specific rates of DALYs for both sexes reached their peak in the 85–89 age group, accompanied by a significant gender disparity (Supplementary Fig. 9). From 1990 to 2021, mortality and DALY rates for male consistently surpassed those for female, especially in 75 + age groups. The peak of age-specific rates of global deaths and DALYs shifted to older age groups over the last three decades regardless of gender (Supplementary Fig. 10 and 11).

The global EAPCs for ASMR and ASDR among males were significantly less than zero, whereas the EAPC for ASMR among females showed the opposite trend, and the ASDR remained stable. The most substantial increases in both ASMR and ASDR were observed among females, with the top three regions being Eastern Sub-Saharan Africa, Southern Latin America, and Central Europe (Supplementary Fig. 4).

## Discussion

This study used the latest GBD 2021 data to assess global trends in the burden of lung cancer attributable to occupational asbestos exposure. We found, for the first time, that the incidence of lung cancer, as well as asbestos-related lung cancer deaths and DALYs reached a turning point approximately 25 years after the complete ban on asbestos use. Over the past three decades, lung cancer deaths and DALYs linked to occupational asbestos exposure have increased significantly worldwide. High SDI regions had a greater burden for lung cancer deaths and DALYs due to occupational asbestos exposure compared to low SDI regions. The proportion of lung cancer deaths and DALYs attributed to occupational asbestos exposure has increased significantly in those aged over 70 years globally. Although the mortality and DALYs rates for lung cancer attributable to occupational exposure to asbestos in male are far higher than those in female, EAPC is significantly higher in female than in male worldwide.

It is important to recognize that the health risks associated with asbestos exposure are influenced by multiple factors. Different types and lengths of asbestos fibers exhibit varying degrees of biopersistence and carcinogenic potential [31, 32]. Crocidolite and amosite are regarded as particularly potent carcinogens, while chrysotile is generally considered to pose a comparatively lower cancer risk [33]. In addition, the type of asbestos-containing material and the specific occupational context play a critical role in determining exposure levels. The highest concentrations of asbestos fibers have been documented among workers involved in asbestos textiles industry, insulation-related industries and shipyards, where amosite was often the predominant fiber type [34]. In contrast, construction workers and automobile mechanics have been found to have significantly lower median levels of asbestos fiber exposure [35]. Moreover, evidence from a Danish cohort study indicated that the risk of malignant mesothelioma and pleural cancers was not elevated among vehicle mechanics [36].

There are notable regional disparities in disease distribution, with a clear positive correlation between asbestos-related lung cancer burden and SDI, consistent with findings from a previous study [37]. The intensity and duration of exposure vary significantly across sectors and historical periods, which can contribute to differences in asbestos-related disease burdens between regions and countries. Regions such as Australasia, Western Europe, and high-income North America carry a heavier burden of asbestos-related lung cancer, likely due to their industrial history in shipbuilding, asbestos cement production, and oil refining [38–40]. In these regions, asbestos was widely used throughout much of the twentieth century, extending beyond industrial settings to include various household products such as ironing board covers, toasters, hair dryers, and joint compounds [41]. Additionally, countries where crocidolite was predominantly used, such as Australia and South Africa, have experienced a heavy burden of lung cancer and mesothelioma [42, 43]. However, in these regions, deaths and DALYs of lung cancer attributed to occupational asbestos exposure have stabilized, and corresponding ASRs are generally declining, largely due to the implementation of regulatory laws on asbestos use.

A previous study has reported that mesothelioma cases tend to decrease only 20 to 30 years after the complete banning of asbestos, a finding that aligns with our results [44]. The Nordic countries were among the first to implement asbestos bans, and they have experienced a marked drop in lung cancer ASIR, as well as ASMR and ASDR linked to occupational asbestos exposure over the past decade. A Swedish study conducted in the last century suggested that the effects of preventive measures taken in the 1970s to reduce occupational asbestos exposure should be evaluated after more than 30 years [45]. More recently, a Nordic study confirmed that these preventive actions have been beneficial [46]. These findings underscore that even after a comprehensive ban, it takes decades to see a significant reduction in the burden of asbestos-related diseases.

Notably, while there was a marked resurgence in the last two years, the USA has seen a sustained decline in asbestos-related lung cancer burden for approximately two decades. In fact, asbestos use in the USA sharply decreased since the 1970 s, although it wasn't until March 18, 2024, that the Environmental Protection Agency officially enacted a ban on the use, manufacture, and import of chrysotile asbestos and related products [47].

In contrast to developed regions, areas with lower SDI have experienced significant increases in ASMR and ASDR for lung cancer linked to occupational asbestos exposure, although the overall rates remain lower level. Despite asbestos use being reduced by at least 75% from its peak by 1990 in many industrialized countries, consumption continues to rise in some resource-limited regions [48]. For instance, in East Asia, while Japan and South Korea have banned asbestos, China still mines, imports, and consumes large quantities of asbestos, making it the second-largest consumer and third-largest producer of chrysotile globally [49]. China consumed the majority of its produced asbestos internally, and this continued reliance on asbestos is potentially driving an upward trend in asbestos-related cancer burden in the coming years [50, 51].

Furthermore, despite the 2017 ruling by the Federal Supreme Court banning asbestos, the lung cancer ASIR and asbestos-induced lung cancer ASMR and ASDR in Brazil has remained relatively stable. Studies have indicated that asbestos production and export still persist in the state of Goiás, highlighting the continued complexity of global asbestos use and its associated health impacts [52].

Despite the absence of specific attribution data, several countries with the longest-standing asbestos bans have reached a turning point in lung cancer incidence and ASIR in recent years. However, several potential confounding factors must be taken into account when interpreting these trends. First, advancements in tobacco control policies have led to a decline in smoking prevalence across many high-income countries, which may have contributed to shifts in lung cancer incidence [53]. While the synergism between smoking and asbestos in lung cancer causation is well-documented, previous studies have demonstrated that occupational asbestos exposure remains an independent risk factor for lung cancer [9, 54]. Second, air pollution has increasingly been recognized as a significant contributor to lung cancer, particularly fine particulate matter (PM2.5) exposure [55]. In recent decades, global air pollution levels have continued to rise, coinciding with an increasing burden of air pollution-related malignancies, including lung cancer [56].

Nevertheless, despite the influence of these confounding factors, the consistency between our findings on asbestos-related deaths/DALYs and the observed incidence trends provides compelling evidence that asbestos bans have played a critical role in reducing the burden of lung cancer. Future research incorporating more detailed exposure data, air pollution metrics, and smoking history could further refine these estimates and offer a more precise understanding of the true impact of asbestos regulations on lung cancer burden.

While the significant increase in the global number of lung cancer deaths and DALYs attributable to occupational asbestos exposure, their ASRs have declined. Given the concurrent rise in the proportion of deaths and DALYs among individuals over 70 years old, this outcome may be attributed to the ongoing global shift in population age structures toward older age groups. Although lung cancer is primarily a disease of the elderly, our findings reveal that the proportion of lung cancer deaths caused by asbestos exposure in individuals under 70 years old is lower than the proportion of all-cause lung cancer deaths reported in other studies [57]. These differences among age groups are likely due to the 15–20 years latency period associated with asbestos-induced lung cancer, making younger patients less likely to develop the disease as a result of occupational asbestos exposure.

As previously reported, a marked gender difference has been observed, with the male population bearing a significantly higher burden of asbestos-related disease [58, 59]. However, we found that the ASMR attributable to occupational exposure to asbestos among female has risen significantly over the past three decades on a global scale. Additionally, while the male ASDR has shown a declining trend, the ASDR of female has remained relatively stable. This pattern may indicate that occupational asbestos exposure has become a more significant factor in the lung cancer burden among women. The extent of occupational asbestos exposure among women may have been consistently underestimated due to the male dominance in high-risk occupations and the absence of gender-sensitive exposure assessment tools [60]. A study conducted in China also highlights that asbestos exposure among female workers is an issue that should not be overlooked [61].

It is noteworthy that the ASMR for lung cancer attributable to occupational asbestos exposure rose across nearly all SDI regions in 2020 and 2021, aligning with the global pattern of elevated all-cause mortality during the COVID-19 pandemic [62].

Our study also has some limitations. Firstly, although various mathematical models have been utilized to correct the data, there may still exist incompatibility because the data provided by GBD 2021 come from different sites. The relative risks used in the GBD 2021 estimation of asbestos-attributable lung cancer are primarily derived from studies conducted in high-income countries. The extrapolation of estimates to other regions may not fully account for differences in the type of asbestos used, levels of exposure, workplace conditions, protective measures, and diagnostic practices. Secondly, beyond occupational exposure, individuals may also experience indirect asbestos exposure through environmental factors (such as the demolition of old buildings) or domestic exposure [63]. However, data on these forms of exposure are lacking at present. Moreover, smoking and air pollution also have a latency period of 10–30 years in causing lung cancer, making it challenging to attribute the incidence of lung cancer to specific causes [64]. Finally, the current estimates may both underestimate and overestimate the true asbestos-related lung cancer burden. On one hand, lung cancer caused by occupational asbestos exposure may be underrecognized due to the dominant role of smoking as a causal factor [65, 66]. On the other hand, extensive public awareness campaigns regarding the carcinogenicity of asbestos have received greater attention than most other occupational carcinogens [67–69]. Notably, in Europe, almost all officially recognized cases of occupational lung cancer are attributed to asbestos exposure, despite the fact that many other occupational carcinogens are also prevalent [70, 71].

In summary, our data showed that occupational asbestos exposure remains a significant contributor to the global lung cancer burden. We found, approximately 25 years after the full implementation of asbestos bans in 50 countries, that new cases of lung cancer, and asbestos-related lung cancer deaths and DALYs start to decline. There are only 70 countries which have issued a complete ban on asbestos, while China is the second consumer and third producer of global chrysotile, accounting roughly 40% global lung cancer new cases and deaths annually. Therefore, we believe that lung cancer new cases, deaths and DALYs associated with occupational asbestos exposure are expected to increase in upcoming years. Consequently, there is an urgent need for a complete ban on asbestos use worldwide, particularly in regions with low SDI levels.

## Supplementary Information

Supplementary Material 1.

## Data Availability

No datasets were generated or analysed during the current study.
