# Supplementary material for: Global burden of lung cancer attributable to occupational asbestos exposure: 1990 to 2021
Source: Environ Health. 2025 Oct 30;24:84. doi: 10.1186/s12940-025-01217-z (PMC12573932; doi:10.1186/s12940-025-01217-z)
Supplement: Supplementary file 1 — Supplementary Material 1. [file 12940_2025_1217_MOESM1_ESM.pdf]

Supplementary Materials

Supplementary Text 1 Estimation of Lung Cancer and Asbestos-Attributable Burden in GBD 2021
Supplementary Table 1. Symbols and Asbestos Banning Times for 50 Countries Included in Study
Supplementary Table 2. DALYs and Age-Standardized DALY Rates of Asbestos-Related Lung Cancer From 1990 to 2021
Supplementary Table 3. Joinpoint Regression Analysis of ASDR in SDI Regions From 1990 to 2021
Supplementary Table 4. Deaths and Age-Standardized Mortality Rates of Asbestos-Related Lung Cancer Among Countries and Territories in 1990 and 2021, and Temporal Trends From 1990 to 2021
Supplementary Table 5. DALYs and Age-Standardized DALY Rates of Asbestos-Related Lung Cancer Among Countries and Territories in 1990 and 2021, and Temporal Trends From 1990 to 2021
Supplementary Fig. 1. Lung Cancer Deaths (A) and DALYs (B) Attributable to Occupational Asbestos Exposure With Age Composition by SDI Region From 1990 to 2021
Supplementary Fig. 2. Temporal Trends of Age-Standardized Death (A) and DALY (B) Rates for Lung Cancer Attributable to Occupational Asbestos Exposure by Sex and SDI Level
Supplementary Fig. 3. Comparison of Lung Cancer Deaths (A) and DALYs (B) Attributable to Occupational Asbestos Exposure by Age and Region in 1990 and 2021
Supplementary Fig. 4. ASMR (A) and ASDR (B) of Lung Cancer Attributable to Occupational Asbestos Exposure in 2021 and EAPC of ASMR (C) and ASDR (D) From 1990 to 2021 by sex in Global and 21 Regions
Supplementary Fig. 5. ASMR of Lung Cancer Attributable Occupational Asbestos Exposure at Global Level and 21 Regions by SDI From 1990 to 2021
Supplementary Fig. 6. ASDR of Lung Cancer Attributable to Occupational Asbestos Exposure at Global Level and 21 Regions by SDI From 1990 to 2021
Supplementary Fig. 7. Correlation of EAPC in Lung Cancer Deaths and DALYs Attributable to Occupational Asbestos Exposure With Age-Standardized Rates in 1990 and SDI in 2021
Supplementary Fig. 8. Proportion of Age Subgroups of Lung Cancer Deaths (A) and DALYs (B) Attributable to Occupational Asbestos Exposure by Region in 1990 and 2021
Supplementary Fig. 9. Proportion of Age Subgroups of Lung Cancer Deaths (A) and DALYs (B) Attributable to Occupational Asbestos Exposure at Global Level by Sex and Year
Supplementary Fig. 10. Age-Specific Rates of Global Deaths (A) and DALYs (B) of Lung Cancer Attributable to Occupational Asbestos Exposure with 95% UI by Sex in 2021
Supplementary Fig. 11. Comparison of Age-Specific Rates of Death Between Female and Male Patients in 1990 and 2021
Supplementary Fig. 12. Comparison of Age-Specific Rates of DALY Between Female and Male Patients in 1990 and 2021

Supplementary Text 1 Estimation of Lung Cancer and Asbestos-Attributable Burden in GBD 2021

GBD Overview

The GBD project estimates mortality, years of life lost (YLLs), years lived with disability (YLDs), and disability-adjusted life-years (DALYs) by age and sex for 87 risk factors and risk factor combinations for 204 countries and territories annually. The data were divided into 21 regions, considering both epidemiological similarities and geographic proximity[1]. Furthermore, all countries and regions were classified into five levels based on the Socio-demographic Index (SDI). In the GBD 2021 database, the SDI is a composite measure that integrates total fertility rate, educational attainment, and per capita income, ranging from 0 to 1[2].

The 95% uncertainty intervals (UI) of estimate value provided by the GBD study were generated by 1000 draws of the uncertainty distribution with the 2.5th and 97.5th-ordered percentiles[2].

Assessment of Lung Cancer Burden

According to the International Code of Diseases 10th (ICD10), lung cancer cases were identified as codes C33-C34.9, D02.1-D02.3, D14.2-D14.3, and D38.1 for our study[3]. The estimation of the burden of lung cancer in the GBD study follows a specific methodology[2,4]. Firstly, Calculating the lung cancer mortality-to-incidence ratio (MIR) using data sources covering both incidence and mortality, which were matched by cancer, age, sex, year, and location. The GBD 2021 database dropped all MIR that were based on less than 15 cases to minimize the impact of statistical noise resulting from small sample sizes. Secondly, the mortality estimate is derived by multiplying the incidence data collected from above-mentioned sources by the previously estimated MIR. Thirdly, the Cause of Death Ensemble model is employed to determine the cancer-specific mortality of lung cancer. Finally, the estimated MIR is utilized to convert the final lung cancer mortality estimates into incidence estimates[5,6].

Comparative Risk Assessment

The GBD study analyzes the proportion of lung cancer deaths and DALYs attributable to occupational asbestos exposure using the Comparative Risk Assessment (CRA) framework[7]. This methodology enables the quantification of hierarchically organized risks or causes contributing to health outcomes. For occupational asbestos exposure, the theoretical minimum-risk exposure level (TMREL) was assumed to be no exposure. the exposure prevalence was estimated using the asbestos impact ratio (AIR), defined as the ratio of excess deaths from mesothelioma observed in the general population to those in populations heavily exposed to asbestos. The mesothelioma mortality rate was estimated using GBD 2021 cause of death results, while mesothelioma mortality rates in unexposed and high-exposure populations were estimated using established statistical models[8,9]. The resulting asbestos exposure prevalence, derived from the AIR, was then applied in the calculation of population attributable fractions (PAFs) for lung cancer. For all occupational risk factors, including asbestos exposure, the relative risks (RRs) were derived through a systematic review of published meta-analyses, which was last updated for GBD 2016[10]. For the estimation of RR for lung cancer attributable to occupational asbestos exposure, GBD 2021 relied on two key meta-analyses[8,11]. Following this, GBD 2021 calculated the asbestos-attributable burden of lung cancer deaths and DALYs[1,3,12].

It should be noted that the GBD 2021 study does not provide incident case estimates attributable to asbestos exposure. Therefore, we cannot assess the contribution of asbestos exposure to incident cases of lung cancer (also due to smoking and air pollution). As an alternative, we descriptively examined the time trends of all-cause lung cancer incidence and age-standardized incidence rates after asbestos bans.

References

[1] Brauer M, Roth GA, Aravkin AY, Zheng P, Abate KH, Abate YH, et al. Global burden and strength of evidence for 88 risk factors in 204 countries and 811 subnational locations, 1990–2021: a systematic analysis for the Global Burden of Disease Study 2021. The Lancet. 2024 May;403(10440):2162–203.

[2] Ferrari AJ, Santomauro DF, Aali A, Abate YH, Abbafati C, Abbastabar H, et al. Global incidence, prevalence, years lived with disability (YLDs), disability-adjusted life-years (DALYs), and healthy life expectancy (HALE) for 371 diseases and injuries in 204 countries and territories and 811 subnational locations, 1990–2021: a systematic analysis for the Global Burden of Disease Study 2021. The Lancet. 2024 Apr;S0140673624007578.

[3] Naghavi M, Ong KL, Aali A, Ababneh HS, Abate YH, Abbafati C, et al. Global burden of 288 causes of death and life expectancy decomposition in 204 countries and territories and 811 subnational locations, 1990–2021: a systematic analysis for the Global Burden of Disease Study 2021. The Lancet. 2024 Apr;S0140673624003672.

[4] Global Burden of Disease 2019 Cancer Collaboration, Kocarnik JM, Compton K, Dean FE, Fu W, Gaw BL, et al. Cancer Incidence, Mortality, Years of Life Lost, Years Lived With Disability, and Disability-Adjusted Life Years for 29 Cancer Groups From 2010 to 2019: A Systematic Analysis for the Global Burden of Disease Study 2019. JAMA Oncol. 2022 Mar 1;8(3):420.

[5] Wen YF, Chen MX, Yin G, Lin R, Zhong YJ, Dong QQ, et al. The global, regional, and national burden of cancer among adolescents and young adults in 204 countries and territories, 1990–2019: a population-based study. J Hematol Oncol. 2021 Dec;14(1):89.

[6] Yang X, Zhang T, Zhang H, Sang S, Chen H, Zuo X. Temporal trend of gastric cancer burden along with its risk factors in China from 1990 to 2019, and projections until 2030: comparison with Japan, South Korea, and Mongolia. Biomark Res. 2021 Dec;9(1):84.

[7] Murray CJ, Lopez AD. Global mortality, disability, and the contribution of risk factors: Global Burden of Disease Study. The Lancet. 1997 May;349(9063):1436–42.

[8] Goodman M, Morgan RW, Ray R, Malloy CD, Zhao K. Cancer in asbestos-exposed occupational cohorts: a meta-analysis. Cancer Causes Control. 1999 Aug;10(5):453–65.

[9] Lin RT, Takahashi K, Karjalainen A, Hoshuyama T, Wilson D, Kameda T, et al. Ecological association between asbestos-related diseases and historical asbestos consumption: an international analysis. The Lancet. 2007 Mar;369(9564):844–9.

[10] GBD 2016 Risk Factors Collaborators. Global, regional, and national comparative risk assessment of 84 behavioural, environmental and occupational, and metabolic risks or clusters of risks, 1990-2016: a systematic analysis for the Global Burden of Disease Study 2016. Lancet. 2017 Sep 16;390(10100):1345–422.

[11] Lenters V, Vermeulen R, Dogger S, Stayner L, Portengen L, Burdorf A, et al. A Meta-analysis of Asbestos and Lung Cancer: Is Better Quality Exposure Assessment Associated with Steeper Slopes of the Exposure–Response Relationships? Environ Health Perspect. 2011 Nov;119(11):1547–55.

[12] Forouzanfar MH, Afshin A, Alexander LT, Anderson HR, Bhutta ZA, Biryukov S, et al. Global, regional, and national comparative risk assessment of 79 behavioural, environmental and occupational, and metabolic risks or clusters of risks, 1990–2015: a systematic analysis for the Global Burden of Disease Study 2015. The Lancet. 2016 Oct;388(10053):1659–724.

Supplementary Tables

Supplementary Table 1. Symbols and Asbestos Banning Times for 50 Countries Included in Study

Countries	Symbol	Year of ban	Countries	Symbol	Year of ban	Countries	Symbol	Year of ban	Countries	Symbol	Year of ban
Algeria	●	2009	Egypt	×	2005	Jordan	▲	2006	Romania	⦿	2009
Argentina	▲	2001	Estonia	⦿	2001	Kuwait	+	1995	Saudi Arabia	●	2008
Australia	+	2002	Finland	●	1993	Latvia	×	2001	Serbia	▲	1998
Austria	×	1990	France	▲	1996	Luxembourg	⦿	2002	South Africa	+	2011
Bahrain	⦿	1996	Germany	+	1993	Macedonia	●	2004	South Korea	×	2008
Belgium	●	1998	Honduras	×	2004	Mauritius	▲	2016	Spain	⦿	2002
Brazil	▲	2017	Iceland	⦿	1983	Monaco	+	2010	Sweden	●	1986
Brunei	+	1994	Iran	●	2021	Mozambique	×	2015	Switzerland	▲	1989
Bulgaria	×	2005	Iraq	▲	2016	Nepal	⦿	1991	Turkey	+	2010
Canada	⦿	2018	Ireland	+	2000	Netherlands	●	2015	Ukraine	×	2022
Chile	●	2001	Israel	×	1984	Norway	▲	1984	United Kingdom	⦿	1999
Colombia	▲	2019	Italy	⦿	1992	Oman	+	2008			
Denmark	+	1985	Japan	●	2012	Poland	×	1997			

Supplementary Table 2. DALYs and Age-Standardized DALY Rates of Asbestos-Related Lung Cancer From 1990 to 2021

Variable	No. (95% UI)				No.(95% CI)
	1990		2021		1990-2021
	DALY, ×10^3	ASDR per 100000	DALY, ×10^3	ASDR per 100000	EAPC of ASDR
Global	2583.96(1813.48 to 3397.82)	66.95(47.27 to 87.64)	3335.71(2319.92 to 4336.16)	39.07(27.17 to 50.73)	-1.63(-1.75 to -1.51)
**SDI**					
Low	7.61(2.63 to 16.22)	3.52(1.22 to 7.33)	16.78(6.23 to 33.59)	3.60(1.36 to 7.05)	0.03(-0.14 to 0.19)
Low-middle	27.82(15.74 to 44.09)	4.88(2.79 to 7.55)	86.18(52.70 to 129.89)	6.29(3.86 to 9.42)	0.93(0.88 to 0.97)
Middle	141.16(87.71 to 209.00)	14.79(9.28 to 21.72)	427.35(271.84 to 631.33)	16.56(10.54 to 24.40)	0.53(0.3 to 0.76)
High-middle	656.76(431.54 to 888.08)	64.64(42.67 to 87.10)	849.19(572.85 to 1162.25)	41.94(28.35 to 57.29)	-1.34(-1.49 to -1.19)
High	1748.29(1252.37 to 2267.51)	152.86(109.16 to 198.71)	1952.53(1389.70 to 2493.56)	86.97(61.83 to 111.37)	-1.72(-1.85 to -1.6)
**Regional**					
Andean Latin America	3.38(2.03 to 5.28)	18.15(10.96 to 28.01)	5.96(3.20 to 9.56)	10.51(5.66 to 16.80)	-2.28(-2.72 to -1.83)
Australasia	57.52(42.79 to 71.42)	235.78(174.44 to 293.71)	64.78(47.86 to 80.79)	113.53(83.45 to 142.91)	-2.48(-2.59 to -2.37)
Caribbean	4.62(2.85 to 6.71)	18.23(11.31 to 26.46)	7.26(4.18 to 11.53)	13.35(7.69 to 21.15)	-0.99(-1.13 to -0.85)
Central Asia	13.88(8.28 to 20.18)	28.83(17.23 to 41.92)	13.71(8.12 to 20.68)	17.12(10.28 to 25.57)	-1.82(-1.98 to -1.65)
Central Europe	67.96(41.46 to 97.66)	43.00(26.23 to 61.85)	147.03(94.51 to 207.16)	63.49(40.66 to 89.72)	1.93(1.67 to 2.19)
Central Latin America	10.75(6.85 to 15.09)	13.96(8.92 to 19.57)	24.20(15.33 to 35.79)	9.90(6.29 to 14.64)	-1.10(-1.26 to -0.94)
Central Sub-Saharan Africa	1.23(0.23 to 3.32)	5.80(1.11 to 15.53)	2.88(0.46 to 8.11)	5.72(1.02 to 16.04)	-0.02(-0.4 to 0.36)
East Asia	135.73(79.89 to 211.56)	17.48(10.38 to 26.74)	474.09(291.32 to 731.35)	22.19(13.39 to 33.96)	1.37(0.99 to 1.74)
Eastern Europe	149.87(92.35 to 213.85)	50.59(31.16 to 72.13)	119.86(72.26 to 180.16)	32.91(19.83 to 49.42)	-1.89(-2.14 to -1.65)
Eastern Sub-Saharan Africa	2.85(0.42 to 7.85)	3.92(0.58 to 10.72)	5.68(0.95 to 14.65)	3.80(0.67 to 9.53)	-0.22(-0.39 to -0.05)
High-income Asia Pacific	102.89(67.18 to 141.47)	51.97(34.12 to 71.28)	314.71(209.52 to 419.64)	57.38(38.11 to 77.40)	0.80(0.5 to 1.09)
High-income North America	653.55(459.34 to 849.89)	177.10(123.71 to 231.36)	587.47(413.60 to 751.61)	82.96(58.28 to 106.19)	-2.81(-3.08 to -2.53)
North Africa and Middle East	89.48(52.51 to 139.93)	53.86(31.86 to 83.34)	133.54(75.47 to 211.87)	31.40(18.02 to 49.57)	-1.85(-2.21 to -1.48)
Oceania	0.27(0.12 to 0.47)	10.14(4.79 to 17.21)	0.65(0.29 to 1.16)	10.17(4.64 to 17.74)	0.14(0.01 to 0.27)
South Asia	20.32(10.42 to 33.00)	3.77(1.95 to 6.09)	67.52(36.54 to 110.18)	4.68(2.53 to 7.57)	0.52(0.36 to 0.68)
Southeast Asia	23.47(13.16 to 37.12)	9.78(5.53 to 15.36)	70.02(39.83 to 110.98)	11.40(6.58 to 17.97)	0.15(0.01 to 0.29)

Southern Latin America	23.20(13.84 to 32.82)	49.57(29.61 to 70.15)	38.17(24.85 to 53.51)	42.78(27.83 to 60.10)	0.13(-0.19 to 0.45)
Southern Sub-Saharan Africa	14.80(9.16 to 21.99)	55.90(35.00 to 82.66)	34.77(22.27 to 48.43)	61.89(40.38 to 85.47)	-0.04(-0.84 to 0.75)
Tropical Latin America	23.61(15.17 to 32.83)	27.08(17.49 to 37.46)	56.62(37.99 to 76.60)	22.45(15.06 to 30.32)	-0.42(-0.52 to -0.33)
Western Europe	1182.01(852.38 to 1511.71)	199.36(143.10 to 256.06)	1162.20(845.14 to 1453.77)	120.70(87.18 to 151.58)	-1.39(-1.51 to -1.27)
Western Sub-Saharan Africa	2.57(1.15 to 4.37)	3.03(1.35 to 5.16)	4.57(2.10 to 8.06)	2.61(1.22 to 4.58)	-0.62(-0.8 to -0.45)

UI, uncertainty interval; CI, confidence interval; DALY, disability-adjusted life years; ASDR, age-standardized DALY rate; EAPC, estimated annual percentage change.

Supplementary Table 3. Joinpoint Regression Analysis of ASDR in SDI Regions From 1990 to 2021

	Both sexes			Male			Female		
	Period	APC/AAPC (95% CI)	*p*-values	Period	APC/AAPC (95% CI)	*p*-values	Period	APC/AAPC (95% CI)	*p*-values
Global	1990~1995	-0.8 (-1.1, -0.3)	0.017	1990~1995	-1.1 (-1.4, -0.5)	0.014	1990~1995	0.7 (0.5, 1.2)	0.020
	1995~1998	-2.4 (-2.7, -1.4)	0.0024	1995~1998	-2.7 (-3.0, -1.8)	< 0.0001	1995~1998	-1.0 (-1.3, 0.3)	0.056
	1998~2007	-1.3 (-1.7, -0.9)	0.00080	1998~2013	-1.4 (-1.5, -1.1)	< 0.0001	1998~2006	0.3 (-0.6, 0.7)	0.076
	2007~2012	-0.7 (-2.0, -0.3)	0.0056	2013~2019	-3.7 (-4.4, -1.5)	< 0.0001	2006~2009	1.7 (0.1, 2.1)	0.036
	2012~2019	-3.3 (-3.8, -0.7)	0.0024	2019~2021	-2.0 (-3.5, -1.3)	< 0.0001	2009~2012	0.4 (-2.0, 1.4)	0.46
	2019~2021	-2.1 (-3.1, -1.5)	< 0.0001				2012~2021	-1.6 (-1.9, -1.3)	< 0.0001
	Full Range	-1.7 (-1.8, -1.7)	< 0.0001	Full Range	-2.0 (-2.0, -1.9)	< 0.0001	Full Range	-0.2 (-0.2, -0.1)	< 0.0001
High SDI	1990~2013	-1.4 (-1.5, -1.3)	< 0.0001	1990~2013	-1.9 (-1.9, -1.8)	< 0.0001	1990~1995	1.3 (0.9, 2.2)	< 0.0001
	2013~2021	-3.3 (-3.6, -3.0)	< 0.0001	2013~2021	-3.6 (-4.0, -3.3)	< 0.0001	1995~1998	-0.6 (-1.0, 0.4)	0.31
							1998~2013	0.7 (0.6, 0.9)	0.023
							2013~2021	-2.1 (-2.4, -1.8)	< 0.0001
	Full Range	-1.9 (-1.9, -1.8)	< 0.0001	Full Range	-2.3 (-2.4, -2.3)	< 0.0001	Full Range	-0.1 (-0.1, 0.0)	0.021
High-middle SDI	1990~1994	1.4 (0.7, 2.2)	0.0016	1990~1994	1.0 (0.3, 1.7)	0.0032	1990~1994	1.0 (0.5, 1.6)	< 0.0001
	1994~1998	-2.8 (-3.6, -1.9)	0.0048	1994~1999	-2.8 (-3.7, -2.2)	< 0.0001	1994~1998	-1.6 (-2.3, -1.0)	< 0.0001
	1998~2006	-1.0 (-1.7, -0.5)	0.011	1999~2012	-0.9 (-1.0, -0.7)	< 0.0001	1998~2007	1.0 (0.8, 1.2)	< 0.0001
	2006~2012	-0.3 (-3.6, 0.5)	0.34	2012~2021	-3.4 (-3.6, -3.2)	< 0.0001	2007~2010	4.0 (2.9, 4.4)	< 0.0001
	2012~2021	-3.1 (-3.5, -2.4)	< 0.0001				2010~2021	-0.8 (-0.9, -0.7)	< 0.0001
	Full Range	-1.4 (-1.5, -1.3)	< 0.0001	Full Range	-1.7 (-1.7, -1.6)	< 0.0001	Full Range	0.3 (0.3, 0.4)	< 0.0001
Low SDI	1990~1997	-0.2 (-0.3, 0.0)	0.047	1990~1997	-0.1 (-0.2, 0.0)	0.068	1990~1999	1.0 (0.6, 1.2)	< 0.0001
	1997~2001	-1.5 (-1.8, -1.0)	0.025	1997~2001	-1.3 (-1.6, -1.0)	0.0076	1999~2003	4.7 (3.9, 5.9)	< 0.0001
	2001~2007	-0.6 (-1.0, -0.2)	0.023	2001~2007	-0.4 (-0.7, -0.2)	0.0064	2003~2010	0.8 (0.3, 1.1)	0.019
	2007~2011	0.7 (-0.2, 1.1)	0.092	2007~2011	0.9 (0.2, 1.2)	0.024	2010~2018	3.7 (3.5, 4.1)	< 0.0001
	2011~2018	1.4 (1.3, 1.8)	< 0.0001	2011~2018	1.5 (1.4, 1.8)	< 0.0001	2018~2021	0.3 (-0.6, 1.2)	0.29
	2018~2021	0.2 (-0.3, 0.6)	0.15	2018~2021	0.5 (0.1, 0.8)	0.035			

	Full Range	0.1 (0.1, 0.1)	< 0.0001	Full Range	0.2 (0.2, 0.2)	< 0.0001	Full Range	2.0 (2.0, 2.1)	< 0.0001
	1990~2003	1.1 (1.0, 1.2)	< 0.0001	1990~2003	1.3 (1.2, 1.4)	< 0.0001	1990~1998	1.1 (0.8, 1.3)	0.00080
	2003~2015	0.7 (0.4, 0.8)	0.012	2003~2015	0.8 (0.3, 0.9)	0.017	1998~2002	2.7 (1.1, 3.3)	< 0.0001
	2015~2019	1.7 (1.3, 2.2)	< 0.0001	2015~2018	2.0 (1.3, 2.4)	< 0.0001	2002~2010	1.8 (1.6, 2.7)	< 0.0001
Low-middle SDI	2019~2021	-1.8 (-2.6, -0.7)	< 0.0001	2018~2021	-0.6 (-1.5, -0.2)	0.018	2010~2013	0.8 (0.4, 2.0)	< 0.0001
							2013~2016	2.9 (0.9, 3.2)	< 0.0001
							2016~2019	1.5 (1.1, 2.4)	< 0.0001
							2019~2021	-1.5 (-2.2, -0.7)	0.0028
	Full Range	0.9 (0.8, 0.9)	< 0.0001	Full Range	1.0 (0.9, 1.0)	< 0.0001	Full Range	1.5 (1.4, 1.5)	< 0.0001
	1990~1997	1.6 (1.3, 2.1)	< 0.0001	1990~1994	1.1 (-0.3, 1.8)	0.11	1990~2007	0.7 (0.6, 0.8)	< 0.0001
	1997~2007	0.7 (0.3, 0.9)	0.013	1994~1997	2.6 (0.2, 3.2)	0.028	2007~2011	4.6 (4.0, 6.0)	< 0.0001
	2007~2011	3.2 (2.6, 4.0)	< 0.0001	1997~2006	0.6 (0.2, 2.9)	0.025	2011~2015	-2.3 (-3.3, -1.6)	< 0.0001
Middle SDI	2011~2018	-2.5 (-2.9, -2.2)	0.0012	2006~2011	2.8 (-2.3, 3.4)	0.085	2015~2021	0.9 (0.4, 1.8)	0.0032
	2018~2021	-0.2 (-1.2, 1.3)	0.71	2011~2018	-2.6 (-3.3, -2.3)	0.0028			
				2018~2021	-0.6 (-1.7, 0.8)	0.26			
	Full Range	0.4 (0.3, 0.5)	< 0.0001	Full Range	0.3 (0.3, 0.4)	< 0.0001	Full Range	0.8 (0.8, 0.9)	< 0.0001

ASDR, age-standardized rate of disability-adjusted life years; SDI, Socio-demographic Index; APC, annual percentage change; AAPC, average annual percentage change; CI, confidence interval.

Supplementary Table 4. Deaths and Age-Standardized Mortality Rates of Asbestos-Related Lung Cancer Among Countries and Territories in 1990 and 2021, and Temporal Trends From 1990 to 2021

Variable	No. (95% UI)				No.(95% CI)
	1990		2021		1990-2021
	Death number	ASMR per 100000	Death number	ASMR per 100000	EAPC of ASMR
Afghanistan	4.33 (1.43 to 11.46)	0.08 (0.03 to 0.20)	5.69 (2.24 to 12.32)	0.09 (0.04 to 0.20)	0.90 (0.70 to 1.11)
Albania	28.65 (11.43 to 48.36)	1.49 (0.62 to 2.49)	63.50 (28.10 to 123.24)	1.36 (0.60 to 2.62)	0.06 (-0.24 to 0.35)
Algeria	6.34 (2.68 to 14.00)	0.11 (0.05 to 0.21)	22.59 (9.11 to 45.94)	0.11 (0.05 to 0.21)	1.17 (0.60 to 1.75)
American Samoa	0.37 (0.20 to 0.62)	2.14 (1.21 to 3.47)	0.27 (0.15 to 0.46)	0.80 (0.44 to 1.31)	-3.16 (-4.10 to -2.23)
Andorra	9.73 (5.71 to 15.18)	16.88 (10.01 to 26.19)	13.56 (7.90 to 20.48)	8.68 (5.04 to 13.16)	-1.99 (-2.36 to -1.63)
Angola	10.20 (1.71 to 26.39)	0.32 (0.06 to 0.85)	33.54 (6.84 to 88.37)	0.36 (0.08 to 0.94)	0.44 (0.24 to 0.63)
Antigua and Barbuda	0.19 (0.11 to 0.28)	0.32 (0.19 to 0.48)	0.19 (0.12 to 0.29)	0.20 (0.12 to 0.31)	-1.49 (-2.03 to -0.96)
Argentina	788.63 (478.37 to 1128.05)	2.45 (1.49 to 3.48)	1493.54 (978.88 to 2061.92)	2.56 (1.68 to 3.55)	0.90 (0.53 to 1.28)
Armenia	105.59 (67.33 to 152.86)	3.94 (2.54 to 5.65)	158.27 (98.15 to 225.38)	3.60 (2.23 to 5.12)	-0.28 (-0.63 to 0.07)
Australia	2330.84 (1763.67 to 2855.60)	11.41 (8.65 to 14.03)	3288.53 (2410.10 to 4062.38)	6.57 (4.82 to 8.14)	-1.85 (-1.95 to -1.75)
Austria	591.08 (399.27 to 791.04)	4.65 (3.13 to 6.21)	705.26 (473.37 to 960.58)	3.62 (2.42 to 4.93)	-0.13 (-0.42 to 0.16)
Azerbaijan	17.80 (7.92 to 32.63)	0.37 (0.17 to 0.64)	29.59 (13.33 to 53.71)	0.31 (0.15 to 0.56)	0.03 (-0.31 to 0.36)
Bahamas	1.66 (0.96 to 2.42)	1.16 (0.67 to 1.68)	2.87 (1.47 to 4.79)	0.79 (0.41 to 1.29)	-1.24 (-1.45 to -1.04)
Bahrain	3.59 (1.99 to 5.54)	3.11 (1.72 to 4.73)	15.05 (7.87 to 24.38)	3.25 (1.80 to 5.06)	0.66 (-0.23 to 1.55)
Bangladesh	113.33 (46.38 to 209.08)	0.27 (0.11 to 0.48)	230.83 (82.03 to 456.54)	0.18 (0.07 to 0.35)	-1.46 (-1.81 to -1.11)
Barbados	1.94 (1.18 to 2.82)	0.58 (0.36 to 0.84)	2.65 (1.44 to 4.23)	0.50 (0.27 to 0.80)	-1.01 (-1.41 to -0.61)
Belarus	241.69 (142.20 to 361.45)	1.81 (1.06 to 2.69)	260.69 (139.76 to 420.38)	1.57 (0.84 to 2.52)	-1.26 (-1.65 to -0.87)
Belgium	2281.39 (1576.67 to 2985.90)	13.90 (9.61 to 18.17)	2211.19 (1596.16 to 2816.02)	8.67 (6.23 to 11.07)	-1.29 (-1.49 to -1.09)
Belize	1.02 (0.62 to 1.45)	1.13 (0.69 to 1.60)	3.20 (1.95 to 4.84)	1.21 (0.75 to 1.83)	-0.63 (-1.53 to 0.27)
Benin	5.36 (2.21 to 10.26)	0.28 (0.12 to 0.54)	8.94 (3.62 to 17.10)	0.20 (0.08 to 0.39)	-0.99 (-1.26 to -0.71)
Bermuda	6.31 (4.59 to 8.04)	10.49 (7.65 to 13.26)	4.67 (2.83 to 7.04)	3.08 (1.86 to 4.69)	-3.59 (-3.95 to -3.22)
Bhutan	0.23 (0.07 to 0.49)	0.11 (0.04 to 0.24)	1.12 (0.51 to 1.98)	0.20 (0.09 to 0.35)	2.19 (2.09 to 2.30)

Bolivia (Plurinational State of)	27.46 (15.46 to 43.75)	1.05 (0.61 to 1.66)	100.75 (53.41 to 171.63)	1.35 (0.73 to 2.29)	1.14 (1.03 to 1.25)
Bosnia and Herzegovina	75.25 (39.53 to 124.01)	1.93 (1.04 to 3.17)	137.62 (66.13 to 235.68)	2.07 (0.99 to 3.53)	0.17 (-0.04 to 0.38)
Botswana	11.17 (5.48 to 18.10)	2.24 (1.12 to 3.59)	22.24 (10.93 to 38.59)	1.86 (0.93 to 3.21)	-2.04 (-2.98 to -1.11)
Brazil	1047.33 (685.92 to 1431.80)	1.39 (0.92 to 1.87)	2998.17 (1985.02 to 4010.38)	1.25 (0.83 to 1.68)	-0.05 (-0.15 to 0.04)
Brunei Darussalam	3.44 (2.02 to 5.33)	4.28 (2.51 to 6.61)	6.18 (3.90 to 9.48)	2.73 (1.74 to 4.02)	-0.35 (-0.85 to 0.14)
Bulgaria	43.19 (22.85 to 70.12)	0.31 (0.17 to 0.51)	78.72 (39.30 to 143.32)	0.53 (0.25 to 0.98)	2.22 (1.90 to 2.55)
Burkina Faso	9.13 (3.13 to 18.56)	0.22 (0.08 to 0.45)	16.03 (5.98 to 31.82)	0.19 (0.07 to 0.38)	-0.27 (-0.56 to 0.02)
Burundi	4.10 (0.70 to 11.50)	0.19 (0.03 to 0.53)	6.49 (0.54 to 18.35)	0.17 (0.02 to 0.47)	-0.71 (-1.03 to -0.38)
Cabo Verde	0.29 (0.11 to 0.54)	0.12 (0.05 to 0.22)	0.88 (0.41 to 1.59)	0.24 (0.11 to 0.44)	0.86 (-0.03 to 1.76)
Cambodia	22.89 (9.31 to 46.68)	0.62 (0.26 to 1.26)	97.03 (40.66 to 186.35)	0.97 (0.43 to 1.83)	1.68 (1.56 to 1.80)
Cameroon	14.11 (5.53 to 25.31)	0.36 (0.14 to 0.64)	32.41 (11.66 to 67.46)	0.31 (0.11 to 0.61)	-0.62 (-0.73 to -0.50)
Canada	3700.99 (2578.43 to 4750.38)	10.96 (7.65 to 14.08)	5031.07 (3711.01 to 6321.71)	6.26 (4.62 to 7.87)	-1.61 (-1.80 to -1.42)
Central African Republic	3.43 (0.68 to 11.24)	0.34 (0.07 to 1.12)	5.45 (0.93 to 16.10)	0.30 (0.05 to 0.88)	-0.42 (-0.47 to -0.36)
Chad	4.68 (1.34 to 9.56)	0.16 (0.05 to 0.33)	11.50 (4.14 to 23.81)	0.24 (0.09 to 0.49)	1.31 (1.19 to 1.42)
Chile	179.11 (116.14 to 250.03)	1.87 (1.22 to 2.61)	340.84 (217.97 to 477.72)	1.29 (0.83 to 1.81)	-0.82 (-0.95 to -0.69)
China	6138.36 (3668.28 to 9428.68)	0.97 (0.59 to 1.47)	25983.83 (15773.56 to 39297.82)	1.36 (0.82 to 2.05)	1.71 (1.30 to 2.13)
Colombia	93.10 (58.47 to 133.63)	0.60 (0.38 to 0.85)	299.41 (160.84 to 494.09)	0.55 (0.30 to 0.91)	-0.31 (-0.57 to -0.06)
Comoros	0.29 (0.04 to 0.82)	0.17 (0.03 to 0.47)	0.91 (0.15 to 2.82)	0.22 (0.04 to 0.66)	0.84 (0.65 to 1.03)
Congo	4.74 (1.24 to 11.11)	0.50 (0.13 to 1.17)	9.03 (2.04 to 23.16)	0.43 (0.11 to 1.10)	-0.68 (-1.03 to -0.33)
Cook Islands	0.11 (0.05 to 0.18)	1.14 (0.57 to 1.85)	0.18 (0.09 to 0.31)	0.76 (0.36 to 1.27)	-1.32 (-1.41 to -1.23)
Costa Rica	6.89 (4.18 to 10.22)	0.43 (0.26 to 0.64)	13.82 (7.82 to 22.49)	0.25 (0.14 to 0.41)	-1.93 (-2.42 to -1.43)
Croatia	215.37 (133.61 to 325.11)	3.49 (2.19 to 5.22)	732.25 (467.50 to 1039.91)	7.62 (4.84 to 10.93)	4.04 (3.15 to 4.93)
Cuba	116.97 (70.02 to 174.77)	1.15 (0.70 to 1.72)	194.44 (108.58 to 300.87)	0.93 (0.52 to 1.45)	-0.51 (-0.80 to -0.23)
Cyprus	56.94 (37.30 to 79.88)	7.93 (5.19 to 11.16)	85.35 (51.30 to 129.08)	3.84 (2.30 to 5.82)	-2.12 (-2.41 to -1.83)
Czechia	472.64 (285.50 to 697.24)	3.29 (1.99 to 4.86)	495.87 (273.61 to 781.58)	2.06 (1.14 to 3.26)	-1.23 (-1.41 to -1.06)
C 么 te d'Ivoire	6.44 (2.43 to 11.46)	0.20 (0.07 to 0.36)	12.56 (4.95 to 25.88)	0.14 (0.06 to 0.28)	-1.89 (-2.16 to -1.62)
Democratic People's	84.37 (42.29 to 158.08)	0.61 (0.32 to 1.07)	209.46 (89.81 to 382.62)	0.65 (0.28 to 1.16)	0.54 (0.34 to 0.74)

Republic of Korea					
Democratic Republic of the Congo	28.91 (4.15 to 90.25)	0.23 (0.04 to 0.70)	64.79 (7.50 to 215.98)	0.22 (0.03 to 0.72)	-0.10 (-0.63 to 0.43)
Denmark	896.91 (617.36 to 1179.22)	10.49 (7.09 to 13.81)	1107.33 (798.23 to 1401.34)	8.23 (5.94 to 10.42)	-0.50 (-0.70 to -0.31)
Djibouti	0.18 (0.02 to 0.54)	0.17 (0.02 to 0.49)	1.61 (0.33 to 4.67)	0.34 (0.08 to 0.97)	2.43 (2.34 to 2.51)
Dominica	1.09 (0.64 to 1.60)	1.76 (1.05 to 2.58)	1.51 (0.79 to 2.44)	1.86 (0.98 to 2.97)	-0.06 (-0.25 to 0.13)
Dominican Republic	6.33 (3.23 to 10.66)	0.22 (0.11 to 0.36)	28.90 (12.89 to 52.74)	0.30 (0.14 to 0.55)	2.06 (1.67 to 2.45)
Ecuador	13.15 (8.32 to 18.67)	0.30 (0.19 to 0.42)	69.03 (41.47 to 105.52)	0.47 (0.29 to 0.70)	2.26 (1.68 to 2.84)
Egypt	6.51 (2.59 to 12.07)	0.04 (0.02 to 0.07)	49.87 (20.09 to 114.92)	0.14 (0.06 to 0.27)	5.57 (4.87 to 6.27)
El Salvador	0.18 (0.03 to 0.42)	0.01 (0.00 to 0.02)	1.64 (0.63 to 3.40)	0.02 (0.01 to 0.05)	5.62 (3.90 to 7.35)
Equatorial Guinea	0.52 (0.09 to 1.50)	0.30 (0.05 to 0.84)	1.61 (0.40 to 4.33)	0.41 (0.10 to 1.08)	1.34 (1.11 to 1.58)
Eritrea	1.38 (0.20 to 4.50)	0.14 (0.02 to 0.46)	5.03 (1.12 to 12.65)	0.23 (0.05 to 0.57)	1.30 (1.03 to 1.58)
Estonia	30.96 (17.66 to 47.23)	1.48 (0.85 to 2.26)	48.71 (27.50 to 75.72)	1.60 (0.89 to 2.52)	0.71 (0.15 to 1.27)
Eswatini	6.76 (2.90 to 14.41)	2.68 (1.15 to 5.64)	14.20 (6.15 to 25.70)	2.84 (1.26 to 5.06)	0.62 (-0.38 to 1.61)
Ethiopia	58.04 (9.36 to 170.30)	0.33 (0.05 to 0.95)	88.14 (12.36 to 227.19)	0.25 (0.04 to 0.65)	-1.01 (-1.41 to -0.61)
Fiji	1.37 (0.68 to 2.54)	0.50 (0.26 to 0.87)	3.99 (1.86 to 7.41)	0.61 (0.30 to 1.08)	1.11 (0.69 to 1.52)
Finland	547.92 (381.99 to 723.42)	7.28 (5.06 to 9.62)	696.65 (478.36 to 906.89)	4.75 (3.28 to 6.21)	-1.15 (-1.28 to -1.01)
France	7353.93 (5076.31 to 9550.57)	8.57 (5.86 to 11.26)	12129.43 (8787.10 to 15078.60)	8.18 (5.86 to 10.24)	0.34 (0.10 to 0.58)
Gabon	2.83 (0.61 to 7.10)	0.52 (0.11 to 1.30)	4.97 (1.35 to 11.85)	0.56 (0.16 to 1.30)	0.12 (-0.01 to 0.25)
Gambia	0.43 (0.16 to 0.86)	0.13 (0.05 to 0.26)	1.04 (0.41 to 2.08)	0.12 (0.05 to 0.24)	-0.74 (-0.97 to -0.51)
Georgia	10.00 (4.36 to 17.09)	0.16 (0.07 to 0.27)	130.77 (68.47 to 212.63)	2.15 (1.12 to 3.51)	13.72 (11.88 to 15.55)
Germany	9975.51 (6874.70 to 13096.17)	7.45 (5.14 to 9.85)	12161.49 (8418.26 to 15640.59)	5.60 (3.89 to 7.23)	-0.57 (-0.73 to -0.41)
Ghana	14.34 (6.50 to 25.67)	0.27 (0.12 to 0.47)	14.92 (6.00 to 28.05)	0.11 (0.04 to 0.20)	-4.61 (-5.68 to -3.54)
Greece	764.45 (476.80 to 1082.37)	4.82 (3.00 to 6.82)	1015.35 (635.69 to 1453.02)	3.84 (2.41 to 5.44)	-1.03 (-1.15 to -0.91)
Greenland	1.34 (0.76 to 2.31)	5.11 (2.89 to 8.59)	7.38 (5.01 to 10.32)	12.44 (8.53 to 17.21)	4.14 (2.81 to 5.46)
Grenada	1.06 (0.69 to 1.49)	1.33 (0.87 to 1.88)	0.97 (0.60 to 1.42)	0.89 (0.56 to 1.28)	-1.11 (-2.30 to 0.07)
Guam	1.48 (0.83 to 2.34)	2.53 (1.52 to 3.85)	1.08 (0.60 to 1.77)	0.44 (0.24 to 0.75)	-5.19 (-5.94 to -4.45)
Guatemala	3.64 (2.25 to 5.35)	0.16 (0.10 to 0.23)	10.83 (6.25 to 17.33)	0.11 (0.06 to 0.18)	-1.85 (-2.25 to -1.45)

Guinea	7.12 (2.23 to 13.86)	0.22 (0.07 to 0.43)	15.03 (5.46 to 31.22)	0.30 (0.11 to 0.61)	1.15 (1.04 to 1.25)
Guinea-Bissau	1.77 (0.73 to 3.59)	0.48 (0.20 to 0.98)	1.94 (0.83 to 3.65)	0.32 (0.14 to 0.60)	-1.12 (-1.50 to -0.73)
Guyana	1.32 (0.81 to 1.96)	0.39 (0.24 to 0.58)	1.99 (1.07 to 3.46)	0.34 (0.19 to 0.59)	-0.20 (-0.59 to 0.18)
Haiti	27.87 (12.17 to 49.17)	1.03 (0.48 to 1.82)	51.30 (22.21 to 103.06)	0.92 (0.41 to 1.78)	0.03 (-0.12 to 0.18)
Honduras	8.50 (3.98 to 13.98)	0.47 (0.23 to 0.77)	64.99 (35.28 to 108.40)	1.17 (0.63 to 1.93)	3.84 (3.47 to 4.20)
Hungary	340.21 (207.53 to 497.47)	2.20 (1.35 to 3.23)	461.92 (259.27 to 735.72)	2.25 (1.26 to 3.60)	0.18 (-0.03 to 0.39)
Iceland	16.86 (11.66 to 21.78)	5.59 (3.86 to 7.23)	28.37 (19.72 to 37.10)	4.53 (3.15 to 5.93)	-0.20 (-0.50 to 0.10)
India	567.72 (303.20 to 939.89)	0.14 (0.08 to 0.24)	2346.76 (1241.01 to 3725.72)	0.21 (0.11 to 0.34)	1.15 (0.92 to 1.38)
Indonesia	204.17 (103.99 to 326.53)	0.26 (0.13 to 0.42)	1045.06 (520.07 to 1780.50)	0.58 (0.29 to 0.98)	2.66 (2.47 to 2.85)
Iran (Islamic Republic of)	9.58 (4.89 to 15.43)	0.06 (0.03 to 0.09)	31.45 (16.83 to 47.72)	0.05 (0.03 to 0.08)	-0.58 (-0.94 to -0.22)
Iraq	80.25 (41.16 to 135.28)	1.13 (0.58 to 1.90)	255.08 (122.67 to 426.67)	1.42 (0.72 to 2.36)	0.10 (-0.12 to 0.33)
Ireland	384.48 (271.32 to 510.38)	8.63 (6.08 to 11.44)	321.91 (217.03 to 433.27)	3.78 (2.54 to 5.10)	-2.45 (-2.66 to -2.24)
Israel	142.61 (92.27 to 200.30)	2.89 (1.87 to 4.06)	283.66 (178.16 to 398.47)	2.16 (1.35 to 3.05)	-0.93 (-1.20 to -0.66)
Italy	10310.08 (7518.03 to 12933.19)	10.96 (7.96 to 13.76)	11571.52 (8323.59 to 14389.40)	6.89 (5.01 to 8.61)	-1.47 (-1.57 to -1.38)
Jamaica	18.66 (11.52 to 28.40)	1.00 (0.62 to 1.52)	22.86 (11.25 to 41.46)	0.75 (0.37 to 1.36)	-1.99 (-2.80 to -1.19)
Japan	5372.82 (3580.68 to 7240.07)	3.23 (2.15 to 4.34)	20085.93 (13449.32 to 26512.12)	4.08 (2.73 to 5.45)	1.23 (0.98 to 1.49)
Jordan	13.06 (7.00 to 20.48)	1.32 (0.74 to 2.05)	47.67 (25.31 to 76.67)	0.88 (0.49 to 1.38)	-1.46 (-1.66 to -1.26)
Kazakhstan	341.67 (202.49 to 501.89)	2.72 (1.61 to 4.02)	171.20 (99.58 to 267.59)	0.96 (0.56 to 1.48)	-4.21 (-4.81 to -3.61)
Kenya	2.99 (0.43 to 8.70)	0.04 (0.01 to 0.12)	18.70 (4.71 to 44.77)	0.10 (0.02 to 0.23)	2.67 (2.35 to 2.99)
Kiribati	0.00 (0.00 to 0.00)	0.05 (0.02 to 0.08)	0.00 (0.00 to 0.01)	0.07 (0.03 to 0.11)	0.97 (0.80 to 1.13)
Kuwait	1.89 (1.06 to 2.93)	0.50 (0.29 to 0.78)	13.45 (7.68 to 20.67)	0.73 (0.42 to 1.13)	2.93 (2.04 to 3.82)
Kyrgyzstan	10.97 (5.83 to 17.60)	0.37 (0.20 to 0.60)	14.40 (7.46 to 25.32)	0.33 (0.17 to 0.57)	0.69 (0.01 to 1.37)
Lao People's Democratic Republic	8.48 (3.32 to 18.51)	0.48 (0.20 to 1.05)	21.19 (8.59 to 43.30)	0.58 (0.25 to 1.15)	0.51 (0.45 to 0.57)
Latvia	68.10 (40.02 to 101.50)	1.84 (1.08 to 2.74)	64.24 (36.19 to 101.12)	1.53 (0.85 to 2.42)	-0.63 (-1.06 to -0.21)
Lebanon	69.97 (41.18 to 111.17)	3.54 (2.09 to 5.54)	197.58 (108.70 to 310.30)	3.15 (1.73 to 4.95)	0.58 (0.03 to 1.13)
Lesotho	13.34 (6.58 to 24.28)	1.64 (0.83 to 2.98)	51.07 (24.60 to 86.13)	4.80 (2.37 to 8.01)	3.89 (3.38 to 4.39)
Liberia	2.80 (1.01 to 5.39)	0.24 (0.09 to 0.46)	2.60 (0.85 to 6.08)	0.16 (0.05 to 0.37)	-1.05 (-1.44 to -0.66)

Libya	4.60 (1.61 to 9.41)	0.30 (0.11 to 0.60)	11.08 (4.37 to 22.34)	0.29 (0.12 to 0.57)	0.30 (0.03 to 0.57)
Lithuania	102.34 (62.44 to 147.75)	2.23 (1.35 to 3.21)	89.73 (51.44 to 136.56)	1.47 (0.84 to 2.25)	-2.14 (-2.49 to -1.78)
Luxembourg	62.88 (44.56 to 79.75)	10.97 (7.70 to 13.94)	69.10 (47.94 to 91.03)	6.26 (4.33 to 8.28)	-1.49 (-1.69 to -1.29)
Madagascar	4.60 (0.30 to 13.10)	0.10 (0.01 to 0.29)	8.54 (0.87 to 25.51)	0.10 (0.01 to 0.29)	0.06 (-0.08 to 0.20)
Malawi	1.84 (0.20 to 4.88)	0.06 (0.01 to 0.15)	5.43 (0.93 to 15.08)	0.09 (0.02 to 0.24)	1.17 (0.91 to 1.43)
Malaysia	24.46 (11.54 to 45.73)	0.32 (0.15 to 0.59)	123.95 (59.43 to 234.41)	0.53 (0.26 to 1.00)	1.55 (1.05 to 2.06)
Maldives	0.47 (0.20 to 0.89)	0.99 (0.45 to 1.77)	1.22 (0.60 to 2.18)	0.49 (0.24 to 0.86)	-3.16 (-3.54 to -2.77)
Mali	6.03 (2.24 to 11.70)	0.17 (0.06 to 0.32)	11.84 (4.15 to 25.75)	0.15 (0.05 to 0.33)	0.06 (-0.25 to 0.38)
Malta	31.82 (21.13 to 41.94)	7.29 (4.84 to 9.63)	52.80 (36.24 to 69.67)	4.81 (3.28 to 6.38)	-0.96 (-1.27 to -0.65)
Marshall Islands	0.14 (0.05 to 0.28)	1.00 (0.41 to 1.93)	0.30 (0.09 to 0.71)	1.25 (0.45 to 2.77)	1.20 (0.89 to 1.51)
Mauritania	2.88 (1.15 to 5.39)	0.28 (0.12 to 0.52)	4.10 (1.59 to 8.17)	0.21 (0.08 to 0.42)	-1.17 (-1.68 to -0.65)
Mauritius	2.44 (1.42 to 3.74)	0.40 (0.23 to 0.61)	2.42 (1.34 to 4.00)	0.15 (0.08 to 0.24)	-3.57 (-4.50 to -2.64)
Mexico	371.46 (240.04 to 519.62)	1.03 (0.67 to 1.43)	722.30 (458.71 to 1063.55)	0.61 (0.39 to 0.90)	-1.60 (-1.82 to -1.39)
Micronesia (Federated States of)	0.45 (0.18 to 0.84)	1.05 (0.45 to 1.88)	0.74 (0.30 to 1.52)	1.24 (0.54 to 2.33)	0.72 (0.61 to 0.82)
Monaco	9.67 (5.88 to 13.91)	12.57 (7.57 to 18.40)	14.86 (9.64 to 20.59)	13.43 (8.70 to 18.68)	0.41 (0.04 to 0.78)
Mongolia	12.82 (6.44 to 23.25)	1.27 (0.64 to 2.27)	17.98 (9.05 to 32.85)	0.90 (0.46 to 1.62)	-1.78 (-2.02 to -1.53)
Montenegro	11.90 (5.58 to 21.63)	1.91 (0.89 to 3.45)	26.93 (13.12 to 50.23)	2.60 (1.29 to 4.80)	1.17 (1.00 to 1.35)
Morocco	15.49 (6.16 to 31.57)	0.13 (0.05 to 0.26)	46.67 (20.34 to 92.57)	0.16 (0.07 to 0.31)	0.91 (0.63 to 1.20)
Mozambique	6.90 (0.73 to 19.05)	0.15 (0.02 to 0.41)	22.87 (5.00 to 57.84)	0.26 (0.06 to 0.66)	2.70 (2.46 to 2.94)
Myanmar	80.61 (28.48 to 181.67)	0.41 (0.15 to 0.89)	181.09 (72.84 to 357.88)	0.44 (0.18 to 0.85)	0.16 (0.08 to 0.25)
Namibia	5.46 (2.95 to 8.26)	0.92 (0.51 to 1.35)	11.37 (6.41 to 18.04)	0.93 (0.54 to 1.45)	-0.54 (-1.06 to -0.02)
Nauru	0.05 (0.02 to 0.09)	1.54 (0.68 to 2.82)	0.05 (0.02 to 0.11)	1.27 (0.54 to 2.43)	-0.63 (-0.86 to -0.40)
Nepal	9.65 (3.81 to 19.29)	0.12 (0.05 to 0.24)	38.93 (17.98 to 71.06)	0.19 (0.09 to 0.34)	1.69 (1.30 to 2.09)
Netherlands	3770.39 (2834.21 to 4557.50)	18.06 (13.53 to 21.86)	3673.64 (2720.26 to 4545.29)	9.40 (6.91 to 11.63)	-2.18 (-2.28 to -2.08)
New Zealand	417.46 (303.74 to 520.03)	10.07 (7.29 to 12.60)	468.95 (345.03 to 589.07)	5.13 (3.76 to 6.47)	-2.13 (-2.29 to -1.98)
Nicaragua	0.73 (0.39 to 1.26)	0.06 (0.03 to 0.10)	2.87 (1.45 to 4.71)	0.07 (0.03 to 0.11)	1.11 (0.76 to 1.46)
Niger	3.77 (1.31 to 8.06)	0.16 (0.06 to 0.34)	9.09 (3.02 to 20.47)	0.13 (0.04 to 0.28)	-0.38 (-0.71 to -0.05)

Nigeria	19.47 (8.27 to 36.09)	0.05 (0.02 to 0.09)	30.96 (12.88 to 62.11)	0.04 (0.02 to 0.08)	-0.03 (-0.18 to 0.12)
Niue	0.01 (0.01 to 0.02)	0.64 (0.32 to 1.10)	0.02 (0.01 to 0.03)	0.89 (0.40 to 1.51)	1.13 (0.94 to 1.31)
North Macedonia	9.74 (5.23 to 16.16)	0.55 (0.30 to 0.90)	21.94 (10.74 to 38.06)	0.65 (0.32 to 1.11)	0.65 (0.37 to 0.93)
Northern Mariana Islands	0.07 (0.03 to 0.13)	0.96 (0.50 to 1.62)	0.28 (0.13 to 0.49)	0.86 (0.44 to 1.37)	0.11 (-0.52 to 0.75)
Norway	468.34 (338.83 to 582.56)	6.32 (4.53 to 7.94)	547.57 (393.91 to 687.02)	4.93 (3.53 to 6.19)	-0.34 (-0.66 to -0.02)
Oman	1.54 (0.62 to 2.91)	0.27 (0.11 to 0.50)	8.27 (4.18 to 14.01)	0.62 (0.34 to 1.01)	4.61 (3.83 to 5.38)
Pakistan	192.41 (76.71 to 345.70)	0.37 (0.15 to 0.67)	498.31 (226.64 to 905.19)	0.50 (0.23 to 0.89)	0.63 (0.28 to 0.98)
Palau	0.07 (0.03 to 0.13)	0.89 (0.41 to 1.55)	0.12 (0.06 to 0.21)	0.89 (0.43 to 1.43)	0.33 (0.20 to 0.46)
Palestine	3.13 (1.44 to 5.78)	0.43 (0.20 to 0.78)	5.29 (2.51 to 9.10)	0.28 (0.13 to 0.46)	-1.48 (-1.71 to -1.25)
Panama	4.07 (2.51 to 5.92)	0.30 (0.18 to 0.44)	9.29 (4.99 to 15.80)	0.21 (0.11 to 0.36)	-1.17 (-1.78 to -0.56)
Papua New Guinea	4.99 (1.58 to 10.23)	0.37 (0.13 to 0.76)	17.36 (5.65 to 36.51)	0.50 (0.18 to 1.03)	1.11 (1.00 to 1.22)
Paraguay	8.34 (4.72 to 13.05)	0.42 (0.24 to 0.65)	67.80 (34.23 to 115.03)	1.29 (0.65 to 2.15)	4.54 (4.25 to 4.84)
Peru	142.12 (86.32 to 220.60)	1.35 (0.82 to 2.08)	186.15 (95.67 to 310.27)	0.58 (0.30 to 0.96)	-3.73 (-4.38 to -3.07)
Philippines	90.84 (52.19 to 138.39)	0.40 (0.23 to 0.60)	215.21 (115.72 to 340.30)	0.30 (0.17 to 0.48)	-1.03 (-1.20 to -0.86)
Poland	886.24 (544.22 to 1290.08)	1.96 (1.20 to 2.84)	3679.04 (2403.87 to 5071.44)	4.79 (3.12 to 6.60)	4.15 (3.66 to 4.64)
Portugal	184.40 (110.71 to 271.89)	1.27 (0.77 to 1.86)	540.82 (333.12 to 772.51)	1.98 (1.22 to 2.85)	2.10 (1.61 to 2.58)
Puerto Rico	41.33 (25.43 to 59.75)	1.16 (0.72 to 1.66)	44.87 (25.34 to 73.34)	0.53 (0.29 to 0.88)	-2.51 (-2.81 to -2.20)
Qatar	0.66 (0.32 to 1.15)	1.18 (0.61 to 1.97)	3.44 (1.55 to 6.47)	0.93 (0.46 to 1.66)	-0.02 (-0.85 to 0.80)
Republic of Korea	273.86 (155.83 to 440.05)	1.11 (0.64 to 1.75)	1636.22 (913.19 to 2490.35)	1.73 (0.97 to 2.63)	1.45 (0.95 to 1.96)
Republic of Moldova	27.06 (15.23 to 42.01)	0.58 (0.33 to 0.91)	19.71 (10.75 to 31.93)	0.32 (0.17 to 0.52)	-2.10 (-2.84 to -1.36)
Romania	245.53 (145.87 to 358.01)	0.82 (0.49 to 1.20)	473.20 (260.56 to 785.00)	1.24 (0.68 to 2.06)	1.19 (0.96 to 1.41)
Russian Federation	3666.61 (2228.37 to 5221.84)	1.94 (1.18 to 2.76)	3893.44 (2419.46 to 5713.22)	1.56 (0.97 to 2.29)	-0.98 (-1.23 to -0.72)
Rwanda	5.77 (1.18 to 14.82)	0.23 (0.05 to 0.60)	12.73 (2.21 to 37.93)	0.25 (0.05 to 0.77)	-0.78 (-1.25 to -0.30)
Saint Kitts and Nevis	0.77 (0.52 to 1.03)	1.94 (1.33 to 2.57)	0.96 (0.59 to 1.44)	1.59 (1.02 to 2.30)	-0.87 (-1.11 to -0.63)
Saint Lucia	0.61 (0.41 to 0.88)	0.80 (0.53 to 1.12)	0.85 (0.49 to 1.33)	0.36 (0.21 to 0.56)	-3.35 (-3.86 to -2.84)
Saint Vincent and the Grenadines	0.36 (0.22 to 0.51)	0.51 (0.32 to 0.72)	0.61 (0.37 to 0.91)	0.45 (0.28 to 0.67)	-0.29 (-0.46 to -0.12)
Samoa	0.27 (0.10 to 0.54)	0.35 (0.13 to 0.67)	0.38 (0.15 to 0.74)	0.30 (0.13 to 0.57)	-0.35 (-0.42 to -0.29)

San Marino	2.70 (1.40 to 4.45)	7.15 (3.66 to 11.80)	3.10 (1.45 to 5.16)	3.65 (1.62 to 6.22)	-0.95 (-1.37 to -0.52)
Sao Tome and Principe	0.25 (0.07 to 0.48)	0.39 (0.11 to 0.76)	0.46 (0.18 to 0.86)	0.51 (0.20 to 0.95)	0.81 (0.66 to 0.95)
Saudi Arabia	0.11 (0.01 to 0.37)	0.00 (0.00 to 0.01)	2.01 (0.38 to 4.29)	0.02 (0.00 to 0.03)	8.98 (7.09 to 10.87)
Senegal	10.15 (4.00 to 19.79)	0.33 (0.13 to 0.65)	21.20 (8.48 to 43.05)	0.30 (0.13 to 0.62)	-0.35 (-0.66 to -0.04)
Serbia	210.37 (117.14 to 328.01)	1.74 (0.97 to 2.72)	361.11 (179.54 to 624.23)	2.07 (1.03 to 3.56)	0.78 (0.45 to 1.10)
Seychelles	0.35 (0.18 to 0.57)	0.61 (0.33 to 1.01)	0.38 (0.20 to 0.64)	0.37 (0.19 to 0.62)	-1.91 (-2.18 to -1.64)
Sierra Leone	5.33 (2.00 to 10.55)	0.26 (0.10 to 0.51)	6.42 (2.37 to 12.85)	0.19 (0.07 to 0.38)	-0.77 (-1.11 to -0.43)
Singapore	116.82 (79.48 to 155.53)	6.31 (4.34 to 8.37)	175.06 (114.83 to 246.28)	2.18 (1.44 to 3.07)	-3.52 (-3.74 to -3.30)
Slovakia	127.98 (74.62 to 204.94)	2.05 (1.20 to 3.29)	125.72 (64.02 to 214.39)	1.24 (0.63 to 2.12)	-1.47 (-1.64 to -1.29)
Slovenia	102.78 (62.75 to 149.97)	4.04 (2.47 to 5.91)	267.14 (160.66 to 396.24)	5.67 (3.38 to 8.48)	1.70 (1.07 to 2.34)
Solomon Islands	0.57 (0.20 to 1.18)	0.53 (0.21 to 1.04)	1.26 (0.50 to 2.59)	0.50 (0.22 to 0.94)	-0.26 (-0.37 to -0.15)
Somalia	2.26 (0.13 to 7.88)	0.12 (0.01 to 0.41)	6.06 (0.42 to 19.73)	0.12 (0.01 to 0.38)	0.38 (0.25 to 0.50)
South Africa	577.82 (365.09 to 833.71)	3.02 (1.92 to 4.28)	1409.15 (918.11 to 1940.41)	3.33 (2.21 to 4.53)	-0.03 (-0.77 to 0.72)
South Sudan	4.13 (0.44 to 11.94)	0.17 (0.02 to 0.47)	6.41 (0.98 to 18.12)	0.22 (0.04 to 0.58)	0.87 (0.76 to 0.97)
Spain	2680.29 (1679.05 to 3751.67)	4.65 (2.91 to 6.53)	4495.65 (2921.85 to 6089.79)	4.25 (2.79 to 5.78)	0.04 (-0.18 to 0.25)
Sri Lanka	42.30 (22.77 to 68.59)	0.51 (0.27 to 0.80)	84.32 (34.83 to 162.35)	0.34 (0.14 to 0.64)	-0.85 (-1.17 to -0.54)
Sudan	5.04 (1.84 to 11.24)	0.07 (0.03 to 0.15)	12.36 (4.83 to 28.03)	0.09 (0.04 to 0.20)	0.82 (0.69 to 0.95)
Suriname	0.66 (0.39 to 1.03)	0.30 (0.17 to 0.46)	2.35 (1.13 to 4.03)	0.39 (0.19 to 0.67)	1.66 (1.03 to 2.28)
Sweden	721.90 (520.87 to 928.53)	4.36 (3.12 to 5.67)	866.80 (600.05 to 1133.63)	3.33 (2.29 to 4.38)	-0.56 (-0.74 to -0.39)
Switzerland	748.13 (512.28 to 968.84)	6.93 (4.69 to 9.04)	990.98 (709.42 to 1254.56)	5.00 (3.55 to 6.37)	-0.69 (-1.00 to -0.39)
Syrian Arab Republic	4.63 (1.88 to 8.85)	0.11 (0.04 to 0.21)	11.83 (4.28 to 24.55)	0.12 (0.05 to 0.24)	0.08 (-0.16 to 0.31)
Taiwan (Province of China)	121.71 (71.31 to 184.58)	0.83 (0.49 to 1.26)	464.81 (251.50 to 768.33)	1.07 (0.58 to 1.76)	0.64 (0.37 to 0.92)
Tajikistan	16.34 (7.54 to 28.71)	0.64 (0.29 to 1.11)	18.73 (8.56 to 36.81)	0.40 (0.19 to 0.77)	-0.97 (-1.37 to -0.57)
Thailand	357.13 (173.00 to 635.06)	1.29 (0.63 to 2.27)	961.82 (421.24 to 1803.15)	0.91 (0.40 to 1.69)	-2.18 (-2.50 to -1.87)
Timor-Leste	0.47 (0.18 to 0.89)	0.24 (0.10 to 0.46)	2.93 (1.31 to 5.53)	0.41 (0.19 to 0.76)	2.03 (1.81 to 2.24)
Togo	3.13 (1.16 to 5.96)	0.29 (0.11 to 0.56)	8.57 (3.20 to 17.99)	0.26 (0.10 to 0.54)	-0.61 (-0.73 to -0.49)
Tokelau	0.01 (0.00 to 0.01)	0.61 (0.25 to 1.08)	0.01 (0.00 to 0.02)	0.64 (0.29 to 1.10)	0.22 (0.06 to 0.38)
Tonga	0.41 (0.16 to 0.73)	0.92 (0.39 to 1.57)	0.68 (0.31 to 1.25)	0.93 (0.42 to 1.68)	-0.09 (-0.43 to 0.25)

Trinidad and Tobago	4.84 (2.99 to 7.17)	0.62 (0.39 to 0.91)	7.72 (3.55 to 14.18)	0.40 (0.19 to 0.73)	-1.71 (-1.95 to -1.46)
Tunisia	8.06 (3.25 to 15.90)	0.20 (0.09 to 0.38)	20.52 (5.96 to 53.71)	0.17 (0.05 to 0.44)	-0.96 (-1.12 to -0.80)
Turkey	3480.40 (2085.59 to 5386.45)	10.87 (6.52 to 16.75)	5411.71 (3029.72 to 8672.66)	5.84 (3.32 to 9.28)	-2.07 (-2.50 to -1.65)
Turkmenistan	5.90 (3.20 to 9.49)	0.31 (0.17 to 0.51)	11.00 (5.22 to 20.25)	0.29 (0.14 to 0.52)	0.02 (-0.37 to 0.42)
Tuvalu	0.04 (0.02 to 0.09)	0.70 (0.31 to 1.44)	0.06 (0.03 to 0.12)	0.75 (0.34 to 1.35)	0.23 (0.16 to 0.29)
Uganda	6.35 (0.46 to 17.76)	0.11 (0.01 to 0.30)	16.48 (2.67 to 46.67)	0.14 (0.02 to 0.38)	0.06 (-0.22 to 0.34)
Ukraine	1661.43 (1038.60 to 2352.93)	2.21 (1.38 to 3.12)	885.36 (449.44 to 1473.23)	1.11 (0.56 to 1.85)	-3.00 (-3.33 to -2.66)
United Arab Emirates	0.35 (0.11 to 0.92)	0.15 (0.05 to 0.35)	1.12 (0.29 to 3.01)	0.12 (0.05 to 0.24)	1.13 (0.53 to 1.73)
United Kingdom	15782.89 (12135.22 to 18906.33)	16.21 (12.40 to 19.47)	13081.92 (10076.20 to 15588.71)	8.90 (6.85 to 10.64)	-1.79 (-1.88 to -1.69)
United Republic of Tanzania	15.29 (1.97 to 42.61)	0.16 (0.02 to 0.43)	42.57 (6.86 to 115.38)	0.20 (0.03 to 0.52)	0.60 (0.52 to 0.67)
United States of America	29949.47 (21302.92 to 38681.88)	8.70 (6.17 to 11.27)	30549.99 (21629.94 to 39075.91)	4.81 (3.41 to 6.16)	-2.25 (-2.53 to -1.97)
United States Virgin Islands	1.32 (0.76 to 2.07)	1.81 (1.07 to 2.83)	3.61 (2.05 to 5.56)	1.85 (1.06 to 2.85)	0.44 (0.21 to 0.67)
Uruguay	119.45 (74.25 to 170.70)	2.96 (1.84 to 4.23)	177.99 (114.06 to 254.47)	2.94 (1.87 to 4.22)	0.23 (-0.01 to 0.47)
Uzbekistan	18.24 (10.20 to 28.49)	0.17 (0.10 to 0.26)	39.25 (23.46 to 63.21)	0.17 (0.10 to 0.27)	0.47 (-0.35 to 1.29)
Vanuatu	0.30 (0.10 to 0.59)	0.65 (0.23 to 1.23)	0.99 (0.38 to 1.90)	0.78 (0.33 to 1.47)	0.72 (0.55 to 0.89)
Venezuela (Bolivarian Republic of)	38.31 (24.70 to 54.30)	0.44 (0.28 to 0.62)	121.23 (60.70 to 224.30)	0.43 (0.22 to 0.78)	-0.30 (-0.47 to -0.14)
Viet Nam	184.90 (86.01 to 337.51)	0.48 (0.23 to 0.87)	611.48 (303.77 to 1034.11)	0.67 (0.33 to 1.10)	0.87 (0.69 to 1.06)
Yemen	2.48 (0.77 to 6.31)	0.07 (0.02 to 0.16)	9.67 (3.42 to 21.59)	0.10 (0.04 to 0.20)	1.57 (1.42 to 1.73)
Zambia	4.54 (0.73 to 11.05)	0.19 (0.03 to 0.47)	17.88 (4.33 to 49.79)	0.32 (0.08 to 0.89)	1.45 (1.19 to 1.72)
Zimbabwe	14.89 (6.91 to 27.43)	0.43 (0.21 to 0.75)	28.61 (15.36 to 48.83)	0.52 (0.28 to 0.88)	0.59 (0.13 to 1.05)

UI, uncertainty interval; CI, confidence interval; ASMR, age-standardized mortality rate; EAPC, estimated annual percentage change.

Supplementary Table 5. DALYs and Age-Standardized DALY Rates of Asbestos-Related Lung Cancer Among Countries and Territories in 1990 and 2021, and Temporal Trends From 1990 to 2021

Variable	No. (95% UI)				No.(95% CI)
	1990		2021		1990-2021
	DALY	ASDR per 100000	DALY	ASDR per 100000	EAPC of ASDR
Afghanistan	88.47 (26.49 to 245.37)	1.43 (0.48 to 3.77)	105.51 (37.24 to 254.49)	1.52 (0.58 to 3.42)	0.61 (0.43 to 0.79)
Albania	660.73 (241.12 to 1123.92)	32.54 (12.41 to 55.01)	1301.99 (558.26 to 2537.59)	27.77 (11.83 to 53.79)	-0.17 (-0.48 to 0.13)
Algeria	113.42 (42.33 to 258.53)	1.44 (0.65 to 3.06)	347.35 (123.00 to 725.06)	1.44 (0.58 to 2.89)	0.68 (0.21 to 1.14)
American Samoa	8.37 (4.50 to 14.45)	41.75 (22.87 to 70.15)	5.13 (2.72 to 8.88)	12.71 (6.90 to 21.46)	-3.85 (-4.97 to -2.74)
Andorra	205.11 (113.00 to 324.21)	346.98 (192.67 to 546.79)	257.16 (142.48 to 404.68)	167.10 (92.17 to 263.16)	-2.21 (-2.56 to -1.87)
Angola	256.43 (39.96 to 692.47)	7.01 (1.18 to 18.19)	802.17 (151.66 to 2132.96)	7.54 (1.56 to 19.84)	0.20 (-0.02 to 0.42)
Antigua and Barbuda	3.29 (1.93 to 4.93)	5.89 (3.45 to 8.84)	3.47 (2.08 to 5.46)	3.42 (2.08 to 5.36)	-1.96 (-2.47 to -1.45)
Argentina	17128.15 (10292.60 to 24722.23)	52.13 (31.30 to 75.07)	28884.14 (18959.57 to 40535.14)	50.57 (33.12 to 70.96)	0.59 (0.19 to 0.99)
Armenia	2755.55 (1713.09 to 4091.80)	95.46 (60.28 to 139.76)	3318.54 (2032.13 to 4837.47)	74.82 (45.76 to 108.89)	-0.92 (-1.24 to -0.59)
Australia	49101.05 (36753.56 to 60926.28)	241.85 (179.76 to 301.29)	56560.04 (41937.91 to 70905.02)	117.59 (86.61 to 148.64)	-2.47 (-2.57 to -2.36)
Austria	11658.90 (7614.38 to 15804.69)	95.75 (62.01 to 129.91)	12848.33 (8552.83 to 17423.75)	70.05 (46.28 to 95.07)	-0.35 (-0.66 to -0.04)
Azerbaijan	467.10 (204.93 to 886.60)	9.11 (4.07 to 16.36)	736.71 (317.32 to 1347.22)	7.07 (3.19 to 12.82)	-0.39 (-0.70 to -0.08)
Bahamas	38.90 (22.42 to 57.46)	25.69 (14.87 to 37.84)	62.13 (31.24 to 106.13)	15.46 (7.77 to 26.15)	-1.59 (-1.79 to -1.38)
Bahrain	76.70 (41.87 to 119.60)	57.52 (31.69 to 88.00)	314.65 (159.27 to 522.68)	52.91 (28.58 to 83.11)	0.12 (-0.71 to 0.95)
Bangladesh	2594.90 (1035.27 to 4765.83)	5.80 (2.36 to 10.48)	4819.10 (1620.92 to 9807.51)	3.55 (1.22 to 7.08)	-1.68 (-1.97 to -1.40)
Barbados	33.35 (20.13 to 48.86)	10.64 (6.43 to 15.37)	45.58 (23.45 to 74.42)	8.61 (4.43 to 14.00)	-1.13 (-1.47 to -0.79)
Belarus	5950.03 (3456.73 to 9013.63)	43.70 (25.44 to 65.89)	5986.13 (3127.21 to 9868.70)	35.88 (18.74 to 59.27)	-1.49 (-1.89 to -1.10)
Belgium	45110.58 (30956.32 to 59567.68)	282.50 (193.89 to 375.47)	39199.04 (28170.91 to 49981.04)	163.92 (117.92 to 210.67)	-1.55 (-1.80 to -1.30)
Belize	20.66 (12.27 to 29.61)	22.70 (13.49 to 32.51)	69.48 (40.83 to 106.73)	24.21 (14.42 to 36.92)	-0.61 (-1.49 to 0.27)
Benin	113.58 (45.70 to 219.10)	5.87 (2.39 to 11.28)	191.62 (77.45 to 369.58)	4.21 (1.68 to 8.14)	-1.03 (-1.32 to -0.75)
Bermuda	127.88 (89.52 to 167.50)	206.43 (145.37 to 269.38)	78.08 (45.94 to 120.76)	54.35 (31.68 to 84.69)	-4.02 (-4.43 to -3.61)
Bhutan	5.43 (1.63 to 12.03)	2.42 (0.76 to 5.19)	21.48 (9.45 to 38.47)	3.72 (1.65 to 6.66)	1.71 (1.60 to 1.83)
Bolivia (Plurinational State	540.91 (298.48 to 878.05)	18.89 (10.69 to 30.22)	1785.85 (921.48 to 3095.58)	22.00 (11.57 to 37.69)	0.75 (0.63 to 0.88)

of)					
Bosnia and Herzegovina	1811.71 (928.61 to 3056.61)	43.51 (22.92 to 71.80)	2912.61 (1330.61 to 4982.37)	44.09 (20.03 to 76.18)	-0.05 (-0.28 to 0.17)
Botswana	264.05 (128.36 to 427.26)	48.13 (23.52 to 77.48)	491.85 (231.07 to 845.51)	37.08 (18.11 to 64.08)	-2.37 (-3.38 to -1.36)
Brazil	23451.29 (15072.12 to 32571.06)	27.60 (17.85 to 38.13)	55358.93 (37203.21 to 74965.99)	22.44 (15.07 to 30.35)	-0.49 (-0.59 to -0.40)
Brunei Darussalam	62.94 (36.30 to 97.82)	74.89 (43.50 to 116.30)	113.22 (68.96 to 178.39)	43.29 (27.32 to 65.93)	-0.89 (-1.31 to -0.46)
Bulgaria	1102.24 (573.85 to 1798.81)	7.98 (4.16 to 13.06)	1877.35 (882.22 to 3487.14)	13.35 (6.09 to 25.22)	2.16 (1.82 to 2.49)
Burkina Faso	202.38 (69.73 to 405.00)	4.73 (1.64 to 9.51)	343.68 (128.29 to 679.68)	3.96 (1.50 to 7.80)	-0.40 (-0.71 to -0.09)
Burundi	94.24 (15.54 to 266.51)	4.14 (0.70 to 11.54)	148.89 (10.50 to 440.80)	3.41 (0.28 to 9.68)	-1.05 (-1.41 to -0.68)
Cabo Verde	5.57 (2.17 to 10.43)	2.31 (0.90 to 4.42)	18.34 (8.10 to 33.86)	4.83 (2.24 to 9.07)	0.89 (-0.01 to 1.79)
Cambodia	546.32 (220.23 to 1106.25)	12.86 (5.28 to 26.06)	2161.67 (861.41 to 4167.18)	18.50 (7.71 to 35.32)	1.38 (1.26 to 1.49)
Cameroon	314.09 (118.23 to 564.24)	7.48 (2.90 to 13.46)	719.61 (255.61 to 1547.97)	6.43 (2.31 to 13.50)	-0.64 (-0.76 to -0.52)
Canada	74452.20 (51214.52 to 97099.87)	221.94 (152.22 to 291.04)	81687.91 (59302.28 to 103542.22)	104.19 (75.36 to 132.38)	-2.31 (-2.51 to -2.11)
Central African Republic	89.53 (17.28 to 293.94)	7.84 (1.59 to 25.59)	143.70 (24.05 to 433.15)	6.74 (1.15 to 19.84)	-0.55 (-0.61 to -0.49)
Chad	98.33 (27.52 to 200.06)	3.43 (0.98 to 6.94)	249.79 (89.83 to 515.90)	4.90 (1.76 to 10.10)	1.26 (1.16 to 1.37)
Chile	3647.78 (2341.13 to 5116.46)	36.98 (23.77 to 51.77)	6066.00 (3832.91 to 8603.88)	23.12 (14.62 to 32.81)	-1.15 (-1.28 to -1.02)
China	131037.95 (77517.98 to 205710.25)	17.61 (10.50 to 27.05)	460853.40 (279094.00 to 712234.13)	22.41 (13.52 to 34.36)	1.40 (1.01 to 1.79)
Colombia	2004.39 (1251.62 to 2887.12)	12.12 (7.59 to 17.43)	5745.75 (2963.57 to 9716.91)	10.52 (5.44 to 17.80)	-0.54 (-0.80 to -0.28)
Comoros	6.79 (0.87 to 20.18)	3.60 (0.50 to 10.23)	18.91 (2.67 to 58.68)	4.21 (0.64 to 13.10)	0.39 (0.17 to 0.62)
Congo	114.19 (28.87 to 268.64)	10.93 (2.86 to 25.95)	212.37 (44.16 to 569.45)	8.76 (1.99 to 22.57)	-0.95 (-1.32 to -0.59)
Cook Islands	2.23 (1.06 to 3.85)	20.30 (9.85 to 33.85)	3.31 (1.51 to 5.71)	12.87 (5.96 to 21.97)	-1.49 (-1.60 to -1.38)
Costa Rica	114.84 (69.51 to 170.64)	7.04 (4.28 to 10.50)	217.47 (116.43 to 357.65)	4.01 (2.15 to 6.62)	-2.10 (-2.61 to -1.58)
Croatia	5161.69 (3155.62 to 7799.04)	79.45 (48.76 to 119.49)	14999.54 (9454.47 to 21649.56)	162.80 (101.68 to 237.16)	3.71 (2.79 to 4.63)
Cuba	2112.28 (1306.34 to 3077.08)	20.57 (12.80 to 30.05)	3528.79 (1975.95 to 5634.89)	17.27 (9.66 to 27.67)	-0.44 (-0.72 to -0.15)
Cyprus	1084.12 (689.78 to 1540.51)	137.32 (87.90 to 195.01)	1503.65 (874.44 to 2317.00)	67.93 (39.21 to 104.71)	-2.03 (-2.33 to -1.72)
Czechia	10659.55 (6385.17 to 15895.00)	75.09 (44.83 to 111.92)	9477.88 (5163.73 to 14957.74)	40.88 (22.15 to 64.99)	-1.73 (-1.90 to -1.57)
C 么 te d'Ivoire	149.48 (57.21 to 265.18)	4.25 (1.61 to 7.59)	285.34 (109.76 to 586.63)	2.91 (1.14 to 5.98)	-1.96 (-2.23 to -1.68)
Democratic People's Republic of Korea	2060.52 (982.06 to 4056.87)	12.97 (6.46 to 24.49)	4836.63 (2029.35 to 8909.24)	14.23 (5.98 to 25.95)	0.63 (0.41 to 0.84)

Democratic Republic of the Congo	691.68 (93.74 to 2158.89)	4.76 (0.70 to 14.89)	1575.04 (157.52 to 5329.96)	4.63 (0.55 to 15.41)	-0.01 (-0.53 to 0.51)
Denmark	18528.63 (12373.34 to 24438.92)	227.43 (150.29 to 300.22)	19213.28 (13818.99 to 24281.22)	149.31 (105.89 to 190.83)	-1.14 (-1.33 to -0.96)
Djibouti	4.36 (0.48 to 13.55)	3.57 (0.44 to 10.85)	37.73 (6.97 to 114.46)	6.76 (1.38 to 19.71)	2.15 (2.07 to 2.24)
Dominica	20.54 (12.24 to 30.45)	33.44 (19.98 to 49.74)	31.08 (15.77 to 51.68)	36.63 (18.72 to 60.57)	0.15 (-0.07 to 0.36)
Dominican Republic	109.10 (53.68 to 179.42)	3.41 (1.72 to 5.58)	495.49 (206.59 to 894.20)	5.10 (2.15 to 9.27)	2.25 (1.85 to 2.65)
Ecuador	227.40 (143.84 to 326.04)	4.80 (3.06 to 6.82)	1037.92 (620.96 to 1641.54)	6.75 (4.06 to 10.54)	1.77 (1.21 to 2.32)
Egypt	135.47 (46.75 to 282.59)	0.65 (0.27 to 1.18)	969.80 (332.26 to 2306.84)	2.13 (0.89 to 4.66)	5.24 (4.61 to 5.86)
El Salvador	2.85 (0.28 to 7.02)	0.10 (0.01 to 0.25)	26.16 (8.68 to 58.00)	0.41 (0.13 to 0.93)	5.89 (3.90 to 7.88)
Equatorial Guinea	12.95 (2.21 to 38.34)	6.74 (1.19 to 19.53)	35.95 (7.56 to 102.10)	8.13 (1.96 to 22.07)	0.80 (0.52 to 1.07)
Eritrea	38.84 (5.27 to 122.39)	3.42 (0.52 to 11.17)	125.92 (26.38 to 322.48)	4.89 (1.11 to 12.34)	0.86 (0.57 to 1.14)
Estonia	718.36 (403.80 to 1120.48)	34.19 (19.26 to 53.03)	863.47 (477.68 to 1367.58)	30.59 (16.72 to 49.03)	-0.02 (-0.56 to 0.53)
Eswatini	162.85 (68.61 to 344.32)	59.60 (25.43 to 126.61)	349.03 (149.56 to 643.70)	63.48 (27.63 to 115.10)	0.59 (-0.49 to 1.67)
Ethiopia	1446.46 (231.11 to 4275.35)	7.42 (1.20 to 21.98)	1768.16 (212.64 to 4596.07)	4.63 (0.60 to 11.89)	-1.75 (-2.19 to -1.31)
Fiji	31.70 (15.40 to 59.60)	10.24 (5.07 to 18.92)	91.02 (41.20 to 171.77)	12.26 (5.78 to 22.65)	1.10 (0.65 to 1.56)
Finland	11190.12 (7663.19 to 14971.96)	151.94 (103.48 to 204.08)	12129.92 (8302.94 to 15863.67)	87.34 (58.93 to 115.38)	-1.57 (-1.72 to -1.41)
France	151382.03 (102149.96 to 203456.24)	184.74 (123.94 to 251.26)	231609.50 (163830.35 to 289681.43)	171.28 (119.69 to 216.67)	0.29 (0.00 to 0.58)
Gabon	63.56 (13.43 to 162.03)	11.12 (2.35 to 28.25)	114.79 (29.29 to 274.23)	11.58 (3.13 to 27.74)	0.04 (-0.08 to 0.16)
Gambia	9.67 (3.56 to 19.30)	2.88 (1.07 to 5.71)	22.78 (8.95 to 45.44)	2.53 (1.01 to 5.10)	-0.81 (-1.04 to -0.57)
Georgia	241.55 (100.98 to 423.33)	3.74 (1.58 to 6.51)	3008.32 (1533.49 to 4946.03)	50.38 (25.58 to 83.13)	13.76 (11.92 to 15.61)
Germany	200658.49 (137096.68 to 266375.56)	155.92 (106.35 to 207.96)	209620.22 (145659.59 to 271099.64)	104.45 (72.59 to 136.80)	-1.04 (-1.20 to -0.88)
Ghana	307.30 (136.31 to 552.54)	5.46 (2.48 to 9.77)	316.09 (128.79 to 602.75)	2.13 (0.86 to 3.99)	-4.67 (-5.75 to -3.59)
Greece	14694.98 (9050.27 to 21063.06)	92.17 (56.66 to 132.20)	17730.14 (11099.99 to 25129.04)	74.79 (46.48 to 106.39)	-0.92 (-1.01 to -0.82)
Greenland	29.73 (16.25 to 51.44)	102.86 (57.81 to 174.41)	151.17 (98.82 to 217.69)	230.87 (157.11 to 324.75)	3.88 (2.55 to 5.21)
Grenada	19.35 (12.57 to 27.50)	26.06 (16.71 to 36.97)	20.00 (12.22 to 29.62)	17.25 (10.76 to 25.29)	-1.03 (-2.02 to -0.04)
Guam	34.60 (19.24 to 53.77)	49.20 (27.97 to 77.10)	20.98 (10.96 to 35.29)	9.15 (4.75 to 15.61)	-4.98 (-5.78 to -4.18)
Guatemala	71.22 (43.22 to 105.15)	2.57 (1.59 to 3.79)	191.10 (106.33 to 317.67)	1.85 (1.04 to 3.04)	-1.64 (-1.97 to -1.32)
Guinea	152.58 (48.17 to 297.26)	4.60 (1.46 to 8.90)	325.20 (114.73 to 684.72)	6.15 (2.20 to 12.96)	1.15 (1.03 to 1.26)

Guinea-Bissau	39.99 (16.11 to 80.56)	10.48 (4.30 to 21.02)	44.63 (18.40 to 85.38)	6.98 (3.00 to 13.13)	-1.14 (-1.52 to -0.77)
Guyana	30.20 (18.31 to 44.82)	8.34 (5.08 to 12.41)	48.09 (24.59 to 86.05)	7.39 (3.91 to 12.91)	-0.11 (-0.51 to 0.29)
Haiti	663.20 (278.23 to 1169.29)	21.54 (9.38 to 38.05)	1155.52 (476.97 to 2354.60)	17.87 (7.75 to 36.13)	-0.24 (-0.41 to -0.08)
Honduras	181.45 (81.92 to 300.44)	9.44 (4.33 to 15.42)	1325.46 (712.94 to 2228.77)	22.16 (11.93 to 37.22)	3.62 (3.28 to 3.97)
Hungary	7976.55 (4890.32 to 11725.43)	52.33 (32.01 to 76.97)	9990.06 (5562.87 to 16090.36)	50.83 (28.17 to 82.81)	-0.01 (-0.22 to 0.20)
Iceland	316.16 (217.06 to 410.77)	107.19 (73.60 to 139.55)	487.03 (339.15 to 643.61)	80.27 (55.85 to 106.53)	-0.47 (-0.75 to -0.18)
India	13396.97 (7041.88 to 22296.66)	3.07 (1.64 to 5.10)	50816.78 (26366.24 to 81037.63)	4.34 (2.26 to 6.89)	0.98 (0.74 to 1.22)
Indonesia	4928.60 (2509.02 to 8011.53)	5.32 (2.70 to 8.52)	23291.14 (11587.91 to 40132.95)	10.73 (5.35 to 18.32)	2.32 (2.13 to 2.51)
Iran (Islamic Republic of)	194.49 (97.31 to 317.84)	0.93 (0.47 to 1.50)	499.14 (262.36 to 758.70)	0.75 (0.40 to 1.14)	-0.91 (-1.28 to -0.55)
Iraq	1647.53 (816.84 to 2831.08)	22.46 (11.18 to 38.37)	5178.46 (2360.28 to 9070.35)	25.59 (12.17 to 43.08)	-0.22 (-0.44 to 0.00)
Ireland	7496.08 (5217.74 to 9998.82)	170.24 (118.07 to 228.38)	5566.86 (3745.98 to 7554.28)	66.60 (44.90 to 90.65)	-2.85 (-3.05 to -2.64)
Israel	2827.21 (1817.63 to 3987.36)	57.40 (36.90 to 80.97)	5003.39 (3114.93 to 7054.14)	39.59 (24.70 to 55.99)	-1.24 (-1.53 to -0.96)
Italy	220250.26 (157970.81 to 279501.35)	239.16 (171.08 to 304.52)	190168.21 (137567.57 to 237746.84)	123.60 (88.45 to 156.20)	-2.14 (-2.23 to -2.05)
Jamaica	362.11 (222.38 to 552.13)	20.03 (12.33 to 30.71)	434.86 (202.76 to 812.33)	14.36 (6.71 to 26.77)	-2.26 (-3.11 to -1.41)
Japan	94676.18 (61948.62 to 129952.13)	55.29 (36.30 to 75.61)	286137.08 (191210.66 to 381427.91)	65.83 (43.13 to 88.15)	1.06 (0.78 to 1.34)
Jordan	281.24 (148.38 to 441.56)	25.08 (13.41 to 39.43)	949.18 (486.86 to 1585.50)	15.36 (8.19 to 24.92)	-1.81 (-2.01 to -1.62)
Kazakhstan	8860.38 (5209.25 to 13087.85)	67.97 (39.92 to 101.31)	4160.02 (2367.07 to 6576.68)	22.07 (12.73 to 34.69)	-4.52 (-5.12 to -3.92)
Kenya	66.75 (8.79 to 188.45)	0.86 (0.12 to 2.47)	431.28 (103.81 to 1043.55)	2.00 (0.50 to 4.77)	2.67 (2.29 to 3.06)
Kiribati	0.02 (0.01 to 0.04)	0.41 (0.18 to 0.65)	0.04 (0.02 to 0.07)	0.58 (0.24 to 0.94)	0.94 (0.78 to 1.11)
Kuwait	36.63 (20.55 to 57.85)	8.87 (4.99 to 13.76)	218.60 (120.62 to 342.14)	11.34 (6.31 to 17.76)	2.51 (1.59 to 3.42)
Kyrgyzstan	276.37 (146.43 to 446.11)	9.11 (4.81 to 14.72)	352.82 (180.90 to 624.67)	7.47 (3.88 to 13.18)	0.42 (-0.25 to 1.09)
Lao People's Democratic Republic	209.11 (79.76 to 454.63)	10.45 (4.08 to 22.33)	469.28 (181.17 to 998.73)	11.02 (4.42 to 22.76)	0.11 (0.05 to 0.17)
Latvia	1673.78 (979.99 to 2531.71)	45.26 (26.47 to 68.48)	1317.01 (728.72 to 2086.17)	33.59 (18.54 to 53.47)	-1.14 (-1.57 to -0.71)
Lebanon	1592.08 (913.99 to 2593.57)	74.33 (42.94 to 119.47)	3652.48 (2015.81 to 5778.93)	60.84 (33.76 to 97.04)	0.29 (-0.26 to 0.84)
Lesotho	303.09 (149.79 to 555.38)	35.90 (17.82 to 65.48)	1280.29 (595.59 to 2210.02)	114.10 (54.29 to 194.80)	4.19 (3.61 to 4.78)
Liberia	59.24 (20.81 to 113.27)	4.92 (1.75 to 9.54)	56.84 (17.83 to 133.23)	3.32 (1.06 to 7.85)	-1.07 (-1.48 to -0.67)
Libya	79.78 (25.38 to 175.83)	4.93 (1.58 to 10.75)	192.30 (67.23 to 408.48)	4.66 (1.79 to 9.61)	0.12 (-0.15 to 0.38)

Lithuania	2364.13 (1429.24 to 3443.44)	51.28 (31.16 to 74.45)	1803.42 (1009.29 to 2765.53)	31.24 (17.24 to 48.16)	-2.42 (-2.78 to -2.06)
Luxembourg	1252.34 (857.42 to 1615.14)	222.27 (150.62 to 288.87)	1250.07 (847.71 to 1670.34)	117.22 (79.17 to 157.12)	-1.81 (-2.00 to -1.62)
Madagascar	105.42 (5.56 to 312.90)	2.19 (0.13 to 6.31)	202.83 (16.57 to 600.05)	2.07 (0.22 to 6.14)	-0.12 (-0.26 to 0.02)
Malawi	42.35 (4.42 to 113.30)	1.17 (0.13 to 3.10)	125.45 (20.06 to 355.66)	1.81 (0.31 to 5.04)	1.15 (0.84 to 1.46)
Malaysia	468.95 (219.97 to 880.22)	5.78 (2.70 to 10.74)	2294.77 (1026.66 to 4379.04)	8.97 (4.16 to 16.89)	1.45 (1.06 to 1.83)
Maldives	9.30 (3.81 to 18.50)	15.13 (6.61 to 27.89)	17.46 (7.98 to 32.38)	6.63 (3.15 to 11.98)	-3.61 (-3.98 to -3.24)
Mali	133.28 (49.70 to 261.24)	3.51 (1.31 to 6.88)	254.58 (87.99 to 571.57)	3.14 (1.09 to 6.89)	-0.02 (-0.35 to 0.31)
Malta	628.12 (410.38 to 835.70)	142.53 (93.06 to 189.58)	949.07 (645.02 to 1268.59)	90.47 (61.44 to 122.09)	-1.10 (-1.39 to -0.81)
Marshall Islands	3.27 (1.24 to 6.63)	21.20 (8.31 to 42.03)	7.14 (2.13 to 17.22)	23.33 (7.59 to 53.52)	0.85 (0.50 to 1.21)
Mauritania	61.54 (23.87 to 116.19)	6.05 (2.45 to 11.07)	86.56 (33.38 to 175.71)	4.28 (1.65 to 8.59)	-1.32 (-1.84 to -0.80)
Mauritius	48.81 (27.75 to 75.41)	7.34 (4.26 to 11.31)	44.76 (23.94 to 74.73)	2.55 (1.39 to 4.27)	-3.85 (-4.76 to -2.93)
Mexico	7442.02 (4769.52 to 10477.57)	19.13 (12.30 to 26.90)	13956.34 (8737.61 to 20729.05)	11.39 (7.16 to 16.90)	-1.64 (-1.87 to -1.42)
Micronesia (Federated States of)	10.18 (4.17 to 19.72)	21.35 (8.89 to 39.93)	17.48 (6.76 to 37.25)	24.42 (9.89 to 49.76)	0.64 (0.54 to 0.74)
Monaco	182.64 (109.12 to 269.37)	257.89 (149.97 to 386.92)	257.52 (164.25 to 359.18)	250.14 (157.96 to 354.08)	0.07 (-0.28 to 0.42)
Mongolia	303.25 (149.29 to 563.34)	29.07 (14.47 to 53.46)	447.36 (216.55 to 834.31)	19.99 (10.01 to 36.69)	-1.85 (-2.10 to -1.60)
Montenegro	283.09 (128.66 to 529.07)	44.23 (20.15 to 81.00)	588.62 (282.37 to 1100.10)	55.99 (26.84 to 104.13)	0.92 (0.69 to 1.15)
Morocco	288.53 (103.16 to 615.86)	2.23 (0.84 to 4.69)	871.37 (351.85 to 1808.05)	2.78 (1.19 to 5.65)	0.85 (0.56 to 1.15)
Mozambique	148.09 (14.90 to 405.48)	2.81 (0.29 to 7.76)	510.02 (107.15 to 1307.86)	5.21 (1.13 to 13.11)	2.84 (2.58 to 3.10)
Myanmar	1953.01 (684.72 to 4545.17)	8.79 (3.13 to 19.91)	3882.81 (1485.49 to 7806.27)	8.37 (3.31 to 16.78)	-0.23 (-0.32 to -0.14)
Namibia	131.22 (70.32 to 204.22)	20.15 (10.96 to 30.83)	262.58 (144.17 to 430.37)	19.87 (11.15 to 31.75)	-0.71 (-1.28 to -0.15)
Nauru	1.12 (0.42 to 2.27)	28.68 (11.87 to 54.89)	1.23 (0.43 to 2.69)	24.23 (9.49 to 49.99)	-0.56 (-0.87 to -0.24)
Nepal	222.33 (85.33 to 448.25)	2.56 (1.01 to 5.07)	799.21 (364.91 to 1467.20)	3.56 (1.65 to 6.50)	1.40 (0.98 to 1.81)
Netherlands	78075.15 (58272.31 to 95617.64)	383.96 (285.07 to 472.16)	65015.77 (47557.88 to 80669.20)	171.94 (125.38 to 214.72)	-2.72 (-2.86 to -2.58)
New Zealand	8419.80 (5997.26 to 10676.19)	205.03 (144.85 to 261.08)	8215.74 (6009.86 to 10400.60)	91.80 (67.14 to 116.37)	-2.59 (-2.76 to -2.42)
Nicaragua	14.66 (7.67 to 26.48)	1.05 (0.55 to 1.88)	56.82 (28.26 to 94.43)	1.23 (0.62 to 2.04)	1.12 (0.75 to 1.48)
Niger	85.23 (29.10 to 180.43)	3.39 (1.18 to 7.25)	197.24 (63.76 to 451.60)	2.58 (0.86 to 5.77)	-0.52 (-0.87 to -0.17)
Nigeria	432.51 (182.68 to 811.13)	0.99 (0.42 to 1.85)	659.80 (268.21 to 1337.21)	0.83 (0.34 to 1.67)	-0.31 (-0.45 to -0.18)

Niue	0.27 (0.13 to 0.48)	12.70 (6.05 to 22.75)	0.36 (0.16 to 0.65)	16.65 (7.22 to 29.53)	0.90 (0.72 to 1.09)
North Macedonia	214.77 (110.96 to 368.07)	11.54 (6.07 to 19.55)	461.38 (221.59 to 837.82)	12.96 (6.44 to 23.37)	0.51 (0.24 to 0.78)
Northern Mariana Islands	1.58 (0.68 to 2.97)	15.65 (7.71 to 27.91)	5.80 (2.56 to 10.41)	13.72 (6.67 to 23.08)	0.13 (-0.55 to 0.81)
Norway	9280.55 (6629.01 to 11711.36)	132.52 (93.36 to 169.41)	9304.31 (6632.49 to 11766.04)	86.64 (61.50 to 110.17)	-0.93 (-1.27 to -0.60)
Oman	35.97 (14.18 to 68.35)	5.89 (2.37 to 11.13)	178.97 (87.52 to 310.60)	11.52 (5.87 to 19.50)	3.96 (3.23 to 4.68)
Pakistan	4098.13 (1593.37 to 7577.58)	7.58 (2.97 to 13.83)	11066.51 (4852.94 to 20965.02)	9.98 (4.51 to 18.43)	0.58 (0.22 to 0.95)
Palau	1.51 (0.63 to 2.85)	16.58 (7.17 to 30.30)	2.79 (1.23 to 4.87)	14.53 (6.81 to 24.28)	-0.26 (-0.37 to -0.15)
Palestine	58.57 (24.92 to 113.44)	7.48 (3.33 to 14.06)	103.64 (45.42 to 189.21)	4.96 (2.32 to 8.56)	-1.41 (-1.62 to -1.21)
Panama	68.85 (42.08 to 100.74)	4.97 (3.04 to 7.26)	144.58 (74.35 to 250.91)	3.29 (1.69 to 5.71)	-1.34 (-1.94 to -0.74)
Papua New Guinea	118.40 (35.93 to 251.32)	7.27 (2.40 to 14.83)	392.86 (124.29 to 838.51)	9.48 (3.14 to 20.04)	0.97 (0.86 to 1.09)
Paraguay	158.41 (88.86 to 257.40)	7.59 (4.29 to 12.26)	1264.11 (640.08 to 2181.87)	22.95 (11.59 to 39.40)	4.34 (4.05 to 4.62)
Peru	2614.99 (1521.70 to 4133.65)	23.95 (14.07 to 37.93)	3132.57 (1556.33 to 5357.79)	9.66 (4.79 to 16.57)	-3.97 (-4.66 to -3.27)
Philippines	2155.19 (1221.96 to 3368.48)	7.74 (4.43 to 11.82)	5082.20 (2610.57 to 8202.32)	6.32 (3.35 to 10.01)	-0.98 (-1.21 to -0.75)
Poland	21322.47 (12923.34 to 31332.87)	46.78 (28.36 to 68.78)	75990.09 (49147.81 to 105510.03)	100.37 (64.81 to 139.66)	3.67 (3.18 to 4.15)
Portugal	3656.53 (2172.55 to 5447.79)	24.89 (14.81 to 37.34)	9636.98 (5853.31 to 13879.92)	38.65 (23.16 to 56.02)	2.11 (1.64 to 2.58)
Puerto Rico	748.23 (461.37 to 1083.51)	20.64 (12.72 to 29.91)	718.42 (392.94 to 1207.80)	9.42 (5.06 to 16.17)	-2.57 (-2.86 to -2.28)
Qatar	14.92 (7.28 to 26.63)	22.15 (10.99 to 37.82)	72.37 (30.26 to 140.70)	15.21 (7.19 to 28.36)	-0.30 (-1.15 to 0.54)
Republic of Korea	5958.07 (3353.26 to 9541.24)	21.77 (12.39 to 35.12)	25646.18 (14208.83 to 39255.83)	26.77 (14.81 to 40.95)	0.78 (0.32 to 1.25)
Republic of Moldova	756.51 (426.46 to 1160.62)	15.90 (8.93 to 24.52)	518.34 (275.84 to 847.39)	8.49 (4.52 to 13.84)	-2.21 (-2.93 to -1.48)
Romania	6579.29 (3892.72 to 9704.84)	21.79 (12.90 to 32.15)	10884.15 (5874.34 to 18211.87)	29.92 (16.25 to 49.83)	0.84 (0.62 to 1.07)
Russian Federation	95634.44 (57678.12 to 136913.40)	49.70 (30.05 to 71.10)	87576.49 (53560.09 to 130336.66)	35.05 (21.43 to 52.17)	-1.51 (-1.79 to -1.24)
Rwanda	139.38 (28.35 to 364.18)	5.06 (1.03 to 13.20)	283.32 (45.67 to 845.07)	4.96 (0.85 to 14.88)	-1.27 (-1.80 to -0.75)
Saint Kitts and Nevis	14.87 (9.89 to 20.24)	38.73 (25.70 to 52.97)	22.12 (13.20 to 33.99)	32.24 (19.76 to 48.36)	-0.91 (-1.21 to -0.60)
Saint Lucia	11.34 (7.51 to 16.21)	13.78 (9.20 to 19.55)	15.33 (8.43 to 24.66)	6.36 (3.52 to 10.22)	-3.14 (-3.59 to -2.69)
Saint Vincent and the Grenadines	6.84 (4.21 to 9.91)	9.67 (6.01 to 13.90)	11.72 (7.10 to 17.69)	8.28 (5.05 to 12.45)	-0.39 (-0.59 to -0.20)
Samoa	6.22 (2.26 to 12.36)	7.34 (2.70 to 14.51)	8.25 (3.28 to 16.59)	6.03 (2.46 to 11.99)	-0.46 (-0.54 to -0.38)
San Marino	49.64 (25.23 to 85.14)	134.85 (67.74 to 232.32)	50.62 (22.13 to 87.33)	65.34 (27.46 to 115.31)	-1.24 (-1.62 to -0.87)

Sao Tome and Principe	5.22 (1.49 to 10.29)	8.02 (2.30 to 15.67)	9.83 (3.81 to 18.82)	10.29 (4.04 to 19.48)	0.74 (0.60 to 0.88)
Saudi Arabia	1.91 (0.11 to 7.17)	0.04 (0.00 to 0.15)	43.92 (7.63 to 95.90)	0.32 (0.06 to 0.70)	10.10 (7.92 to 12.29)
Senegal	221.28 (87.31 to 427.54)	7.05 (2.80 to 13.52)	454.08 (181.87 to 920.58)	6.28 (2.54 to 12.80)	-0.44 (-0.76 to -0.12)
Serbia	5571.14 (3042.85 to 8781.43)	43.63 (24.16 to 68.33)	8416.97 (4162.29 to 14502.46)	49.90 (24.66 to 86.18)	0.57 (0.23 to 0.91)
Seychelles	6.97 (3.55 to 11.83)	12.44 (6.32 to 21.22)	7.84 (3.98 to 13.21)	6.93 (3.51 to 11.64)	-2.17 (-2.45 to -1.90)
Sierra Leone	113.11 (42.17 to 224.56)	5.51 (2.06 to 10.92)	138.42 (51.11 to 282.02)	3.99 (1.46 to 8.07)	-0.80 (-1.15 to -0.45)
Singapore	2190.09 (1465.24 to 2969.83)	112.04 (75.74 to 150.70)	2816.43 (1841.21 to 4018.82)	34.19 (22.38 to 48.62)	-3.90 (-4.13 to -3.68)
Slovakia	3075.63 (1780.11 to 4972.72)	49.57 (28.84 to 80.26)	2770.03 (1404.44 to 4788.05)	27.57 (13.98 to 47.72)	-1.77 (-1.95 to -1.59)
Slovenia	2455.84 (1471.87 to 3643.98)	96.56 (58.01 to 143.37)	5216.70 (3084.54 to 7842.08)	115.87 (68.13 to 175.38)	1.19 (0.54 to 1.85)
Solomon Islands	13.86 (4.66 to 29.80)	10.89 (3.99 to 22.54)	29.85 (10.97 to 62.98)	9.95 (4.03 to 20.14)	-0.35 (-0.50 to -0.21)
Somalia	58.05 (2.94 to 206.76)	2.71 (0.15 to 9.41)	152.33 (10.18 to 500.16)	2.72 (0.19 to 8.63)	0.24 (0.13 to 0.35)
South Africa	13610.30 (8257.88 to 20318.53)	67.16 (41.35 to 99.07)	31726.28 (19681.98 to 44649.06)	69.55 (44.00 to 96.68)	-0.30 (-1.11 to 0.51)
South Sudan	93.72 (9.21 to 276.48)	3.66 (0.38 to 10.53)	147.56 (19.87 to 435.35)	4.35 (0.66 to 12.26)	0.56 (0.41 to 0.71)
Spain	56029.65 (35248.74 to 79522.94)	98.51 (61.83 to 139.97)	80026.12 (52428.98 to 108924.22)	82.19 (53.83 to 112.64)	-0.35 (-0.57 to -0.14)
Sri Lanka	924.75 (483.02 to 1578.98)	9.40 (5.05 to 15.48)	1815.88 (707.72 to 3659.97)	6.78 (2.69 to 13.48)	-0.67 (-1.03 to -0.31)
Sudan	97.91 (31.28 to 235.60)	1.21 (0.44 to 2.74)	220.85 (76.27 to 542.28)	1.42 (0.54 to 3.26)	0.60 (0.47 to 0.73)
Suriname	13.68 (7.84 to 21.92)	5.69 (3.28 to 8.95)	49.85 (23.49 to 86.48)	7.79 (3.64 to 13.41)	1.82 (1.18 to 2.46)
Sweden	13638.24 (9723.79 to 17828.11)	87.58 (61.59 to 116.21)	13546.81 (9396.10 to 17921.26)	54.99 (37.96 to 73.15)	-1.20 (-1.43 to -0.98)
Switzerland	15306.79 (10284.17 to 20126.92)	149.11 (99.64 to 197.24)	17370.98 (12258.50 to 22373.82)	93.74 (65.49 to 121.59)	-1.18 (-1.53 to -0.83)
Syrian Arab Republic	92.36 (34.31 to 183.13)	2.01 (0.79 to 3.83)	236.19 (75.35 to 509.32)	2.04 (0.75 to 4.23)	-0.22 (-0.42 to -0.01)
Taiwan (Province of China)	2636.24 (1532.32 to 4030.38)	16.72 (9.77 to 25.49)	8399.18 (4424.42 to 14061.43)	19.11 (10.09 to 31.98)	0.28 (0.04 to 0.53)
Tajikistan	387.76 (181.38 to 699.39)	14.41 (6.66 to 26.01)	441.95 (199.34 to 897.05)	8.37 (3.81 to 16.33)	-1.30 (-1.68 to -0.93)
Thailand	7789.56 (3572.13 to 14360.56)	23.33 (11.09 to 42.22)	16395.49 (6667.10 to 31852.86)	15.39 (6.33 to 29.84)	-2.41 (-2.74 to -2.08)
Timor-Leste	11.42 (4.00 to 22.61)	4.68 (1.79 to 8.88)	59.98 (26.23 to 113.93)	7.64 (3.37 to 14.75)	1.84 (1.57 to 2.10)
Togo	69.07 (24.78 to 134.92)	6.22 (2.27 to 11.94)	197.04 (72.55 to 408.81)	5.69 (2.13 to 11.91)	-0.55 (-0.67 to -0.42)
Tokelau	0.16 (0.06 to 0.31)	11.19 (4.45 to 21.08)	0.16 (0.07 to 0.29)	10.99 (4.63 to 19.35)	-0.07 (-0.20 to 0.07)
Tonga	8.59 (3.22 to 15.37)	16.83 (6.58 to 29.38)	12.88 (5.45 to 25.00)	16.96 (7.27 to 32.68)	-0.07 (-0.45 to 0.30)
Trinidad and Tobago	97.32 (59.83 to 143.93)	11.95 (7.35 to 17.59)	160.60 (70.06 to 296.96)	8.17 (3.59 to 15.04)	-1.59 (-1.83 to -1.34)

Tunisia	151.66 (52.85 to 305.65)	3.42 (1.35 to 6.65)	370.14 (89.20 to 1022.70)	2.92 (0.76 to 7.88)	-0.89 (-1.04 to -0.75)
Turkey	84379.73 (49459.26 to 133032.92)	245.75 (147.55 to 384.16)	118684.21 (65547.61 to 191654.01)	123.55 (68.23 to 199.23)	-2.33 (-2.77 to -1.90)
Turkmenistan	153.96 (82.90 to 250.00)	7.81 (4.23 to 12.57)	285.13 (132.36 to 535.43)	6.88 (3.27 to 12.69)	-0.14 (-0.54 to 0.26)
Tuvalu	1.03 (0.42 to 2.14)	14.49 (6.09 to 30.00)	1.40 (0.59 to 2.69)	13.91 (6.02 to 25.77)	-0.13 (-0.18 to -0.08)
Uganda	143.82 (9.55 to 414.72)	2.33 (0.16 to 6.65)	372.10 (53.99 to 1080.64)	2.77 (0.43 to 7.89)	-0.17 (-0.50 to 0.16)
Ukraine	42769.81 (26335.90 to 60996.94)	56.77 (34.71 to 80.64)	21797.78 (10766.17 to 36613.70)	27.83 (13.83 to 46.85)	-3.14 (-3.49 to -2.79)
United Arab Emirates	7.50 (1.99 to 21.41)	2.65 (0.85 to 6.83)	26.66 (5.08 to 81.00)	1.74 (0.61 to 3.80)	0.28 (-0.26 to 0.82)
United Kingdom	317540.73 (242341.99 to 382551.94)	339.52 (257.32 to 411.58)	218432.44 (168263.62 to 261059.69)	156.79 (119.90 to 188.12)	-2.40 (-2.48 to -2.33)
United Republic of Tanzania	353.00 (42.22 to 1031.09)	3.33 (0.41 to 9.38)	934.38 (138.26 to 2712.43)	3.98 (0.60 to 11.23)	0.36 (0.28 to 0.44)
United States of America	579051.68 (408523.15 to 756891.48)	172.41 (120.93 to 226.40)	505623.28 (353544.56 to 652553.51)	80.31 (56.08 to 103.70)	-2.90 (-3.19 to -2.61)
United States Virgin Islands	28.95 (16.71 to 46.91)	35.63 (20.77 to 56.14)	66.08 (35.60 to 105.42)	34.42 (18.39 to 55.58)	0.31 (0.09 to 0.53)
Uruguay	2426.19 (1488.29 to 3507.31)	60.70 (37.14 to 88.18)	3218.43 (2019.99 to 4633.65)	57.15 (35.54 to 82.69)	-0.03 (-0.26 to 0.21)
Uzbekistan	435.32 (237.57 to 693.90)	3.92 (2.15 to 6.22)	960.24 (555.35 to 1573.60)	3.72 (2.21 to 5.99)	0.34 (-0.50 to 1.18)
Vanuatu	6.92 (2.14 to 14.26)	12.46 (4.26 to 24.65)	22.61 (8.32 to 43.93)	14.80 (5.79 to 28.17)	0.67 (0.51 to 0.84)
Venezuela (Bolivarian Republic of)	849.23 (541.60 to 1216.99)	9.24 (5.92 to 13.27)	2532.03 (1203.39 to 4785.10)	8.50 (4.11 to 15.98)	-0.55 (-0.72 to -0.38)
Viet Nam	4384.97 (1975.80 to 8184.36)	10.83 (4.94 to 20.14)	14397.04 (7081.99 to 24893.45)	14.24 (7.09 to 24.26)	0.78 (0.64 to 0.92)
Yemen	53.80 (15.30 to 139.22)	1.27 (0.40 to 3.20)	182.25 (59.02 to 435.92)	1.64 (0.57 to 3.68)	1.12 (0.96 to 1.28)
Zambia	103.50 (16.11 to 264.23)	3.95 (0.63 to 9.64)	418.06 (97.52 to 1220.10)	6.65 (1.60 to 18.58)	1.32 (1.03 to 1.61)
Zimbabwe	330.72 (145.77 to 618.84)	8.63 (4.04 to 15.88)	664.74 (340.57 to 1156.28)	10.54 (5.61 to 17.75)	0.53 (0.06 to 1.00)

UI, uncertainty interval; CI, confidence interval; DALY, disability-adjusted life years; ASDR, age-standardized DALY rate; EAPC, estimated annual percentage change.

Supplementary Figures

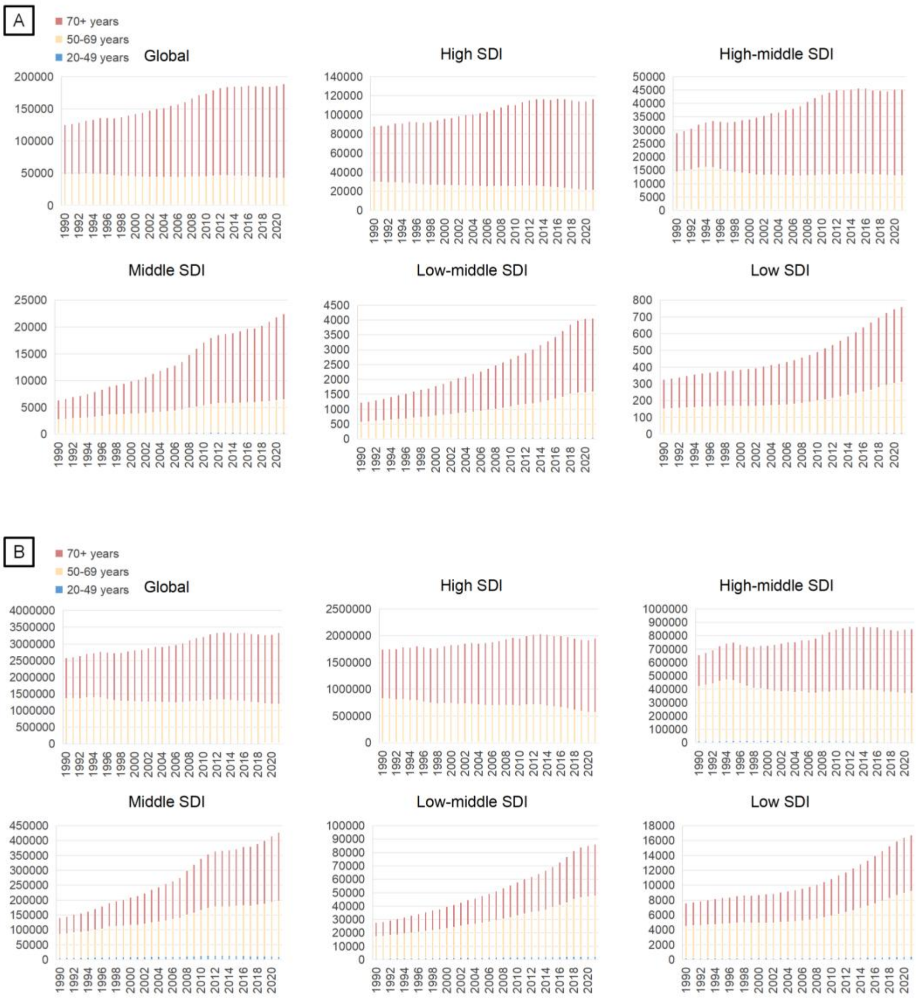

Supplementary Fig. 1. Lung Cancer Deaths (A) and DALYs (B) Attributable to Occupational Asbestos Exposure With Age Composition by SDI Region From 1990 to 2021.

DALY, disability-adjusted life years; SDI, Socio-demographic Index.

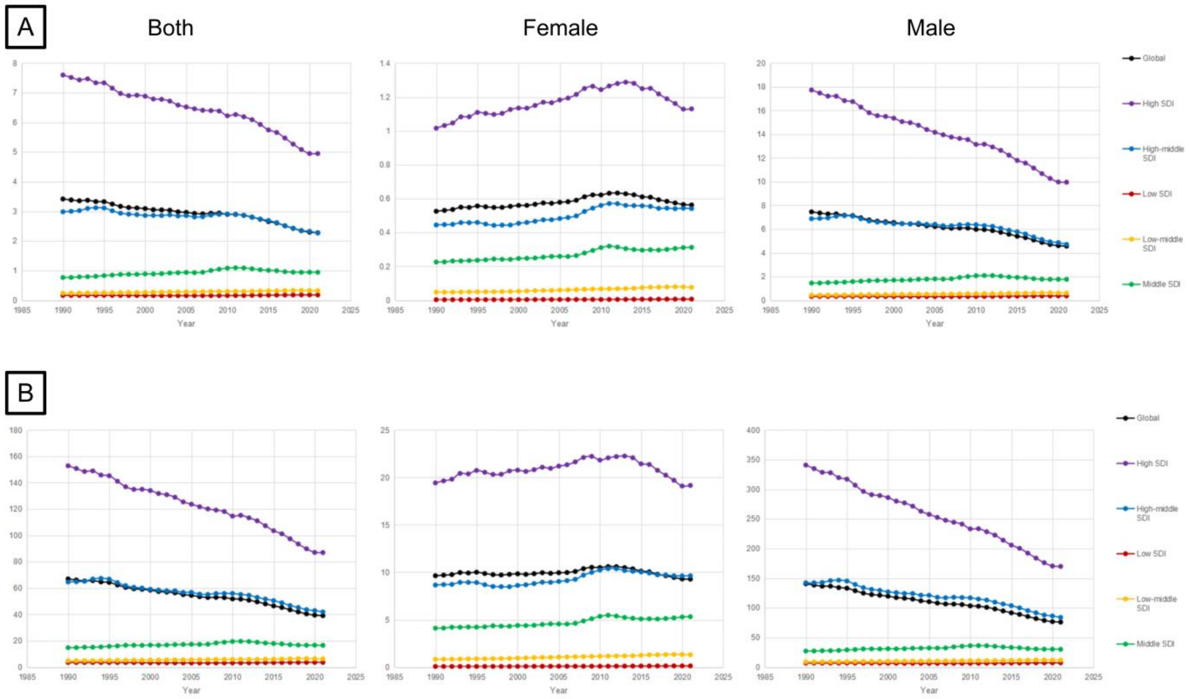

Supplementary Fig. 2. Temporal Trends of Age-Standardized Death (A) and DALY (B) Rates for Lung Cancer Attributable to Occupational Asbestos Exposure by Sex and SDI Level.

DALY, disability-adjusted life years; SDI, Socio-demographic Index.

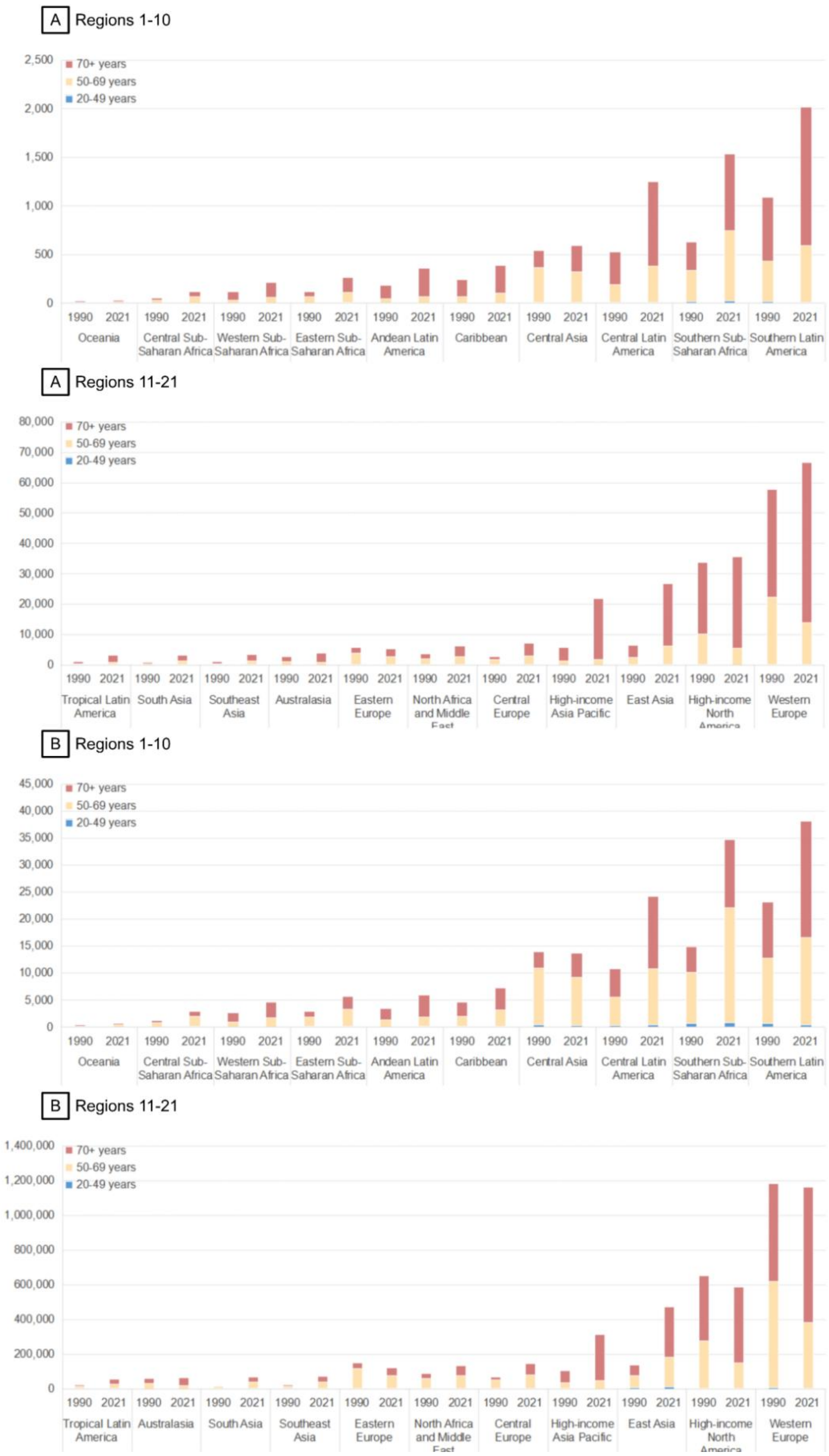

Supplementary Fig. 3. Comparison of Lung Cancer Deaths (A) and DALYs (B) Attributable to Occupational Asbestos Exposure by Age and Region in 1990 and 2021.

DALY, disability-adjusted life years.

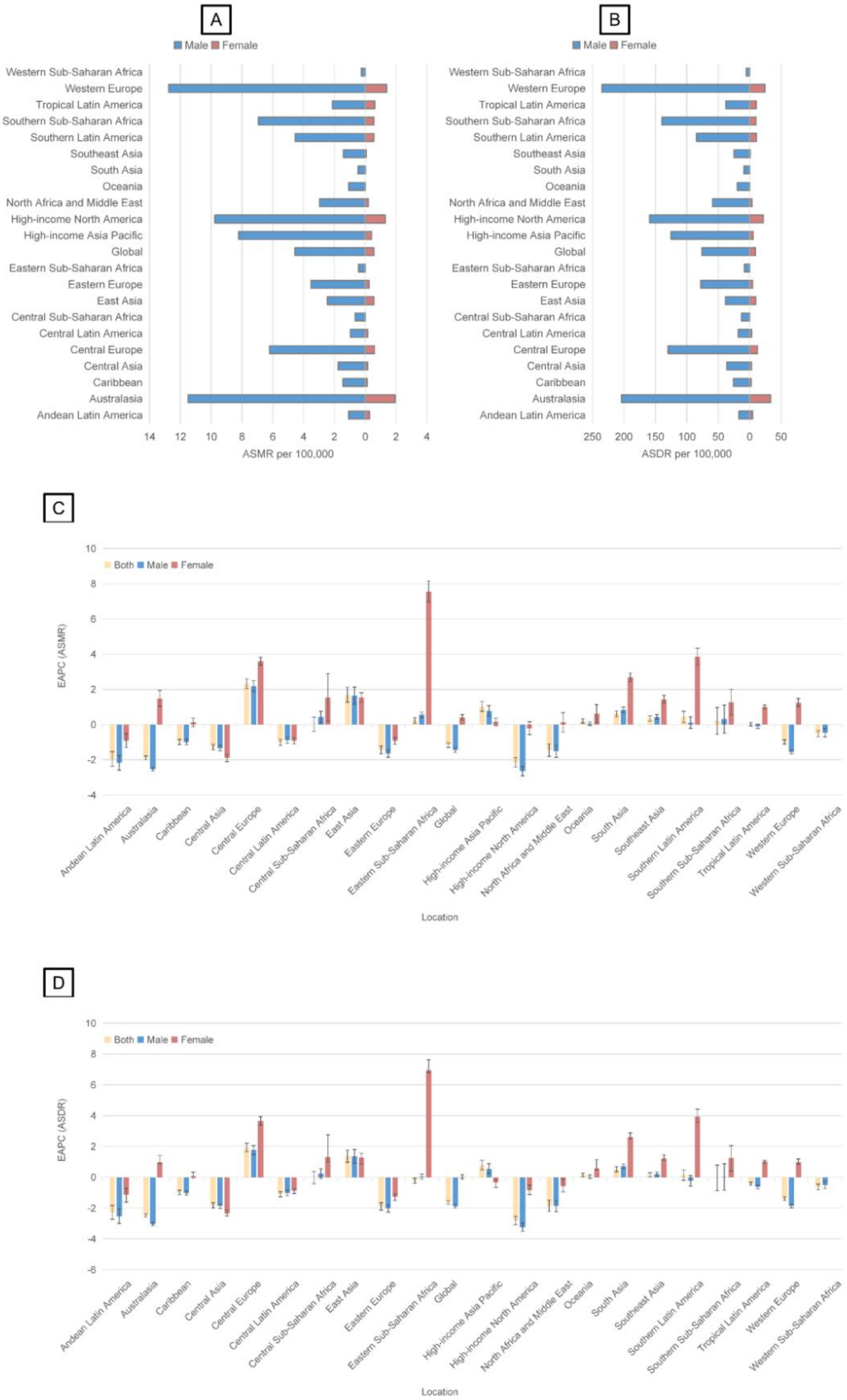

Supplementary Fig. 4. ASMR (A) and ASDR (B) of Lung Cancer Attributable to Occupational Asbestos Exposure in 2021 and EAPC of ASMR (C) and ASDR (D) From 1990 to 2021 by sex in Global and 21 Regions.

ASMR, age-standardized mortality rate; ASDR, age-standardized rate of disability-adjusted life years; EAPC, estimated annual percentage change.

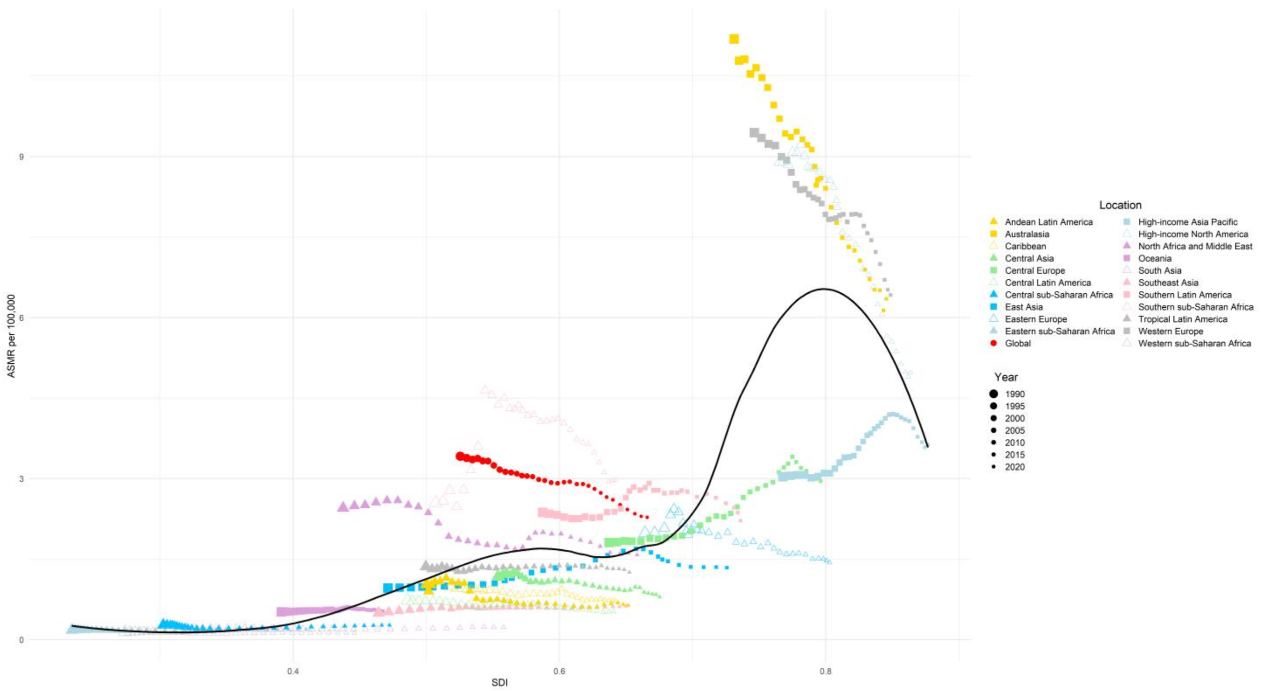

Supplementary Fig. 5. ASMR of Lung Cancer Attributable Occupational Asbestos Exposure at Global Level and 21 Regions by SDI From 1990 to 2021.

ASMR, age-standardized mortality rate; SDI, Socio-demographic Index.

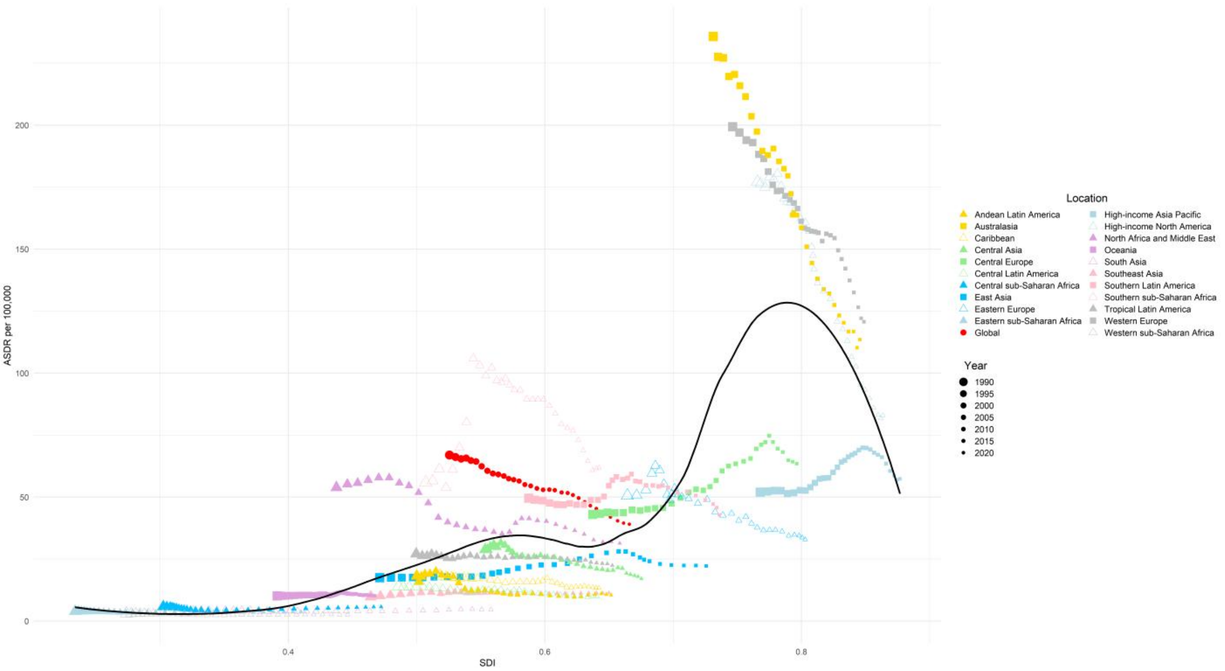

Supplementary Fig. 6. ASDR of Lung Cancer Attributable to Occupational Asbestos Exposure at Global Level and 21 Regions by SDI From 1990 to 2021.

ASDR, age-standardized rate of disability-adjusted life years; SDI, Socio-demographic Index.

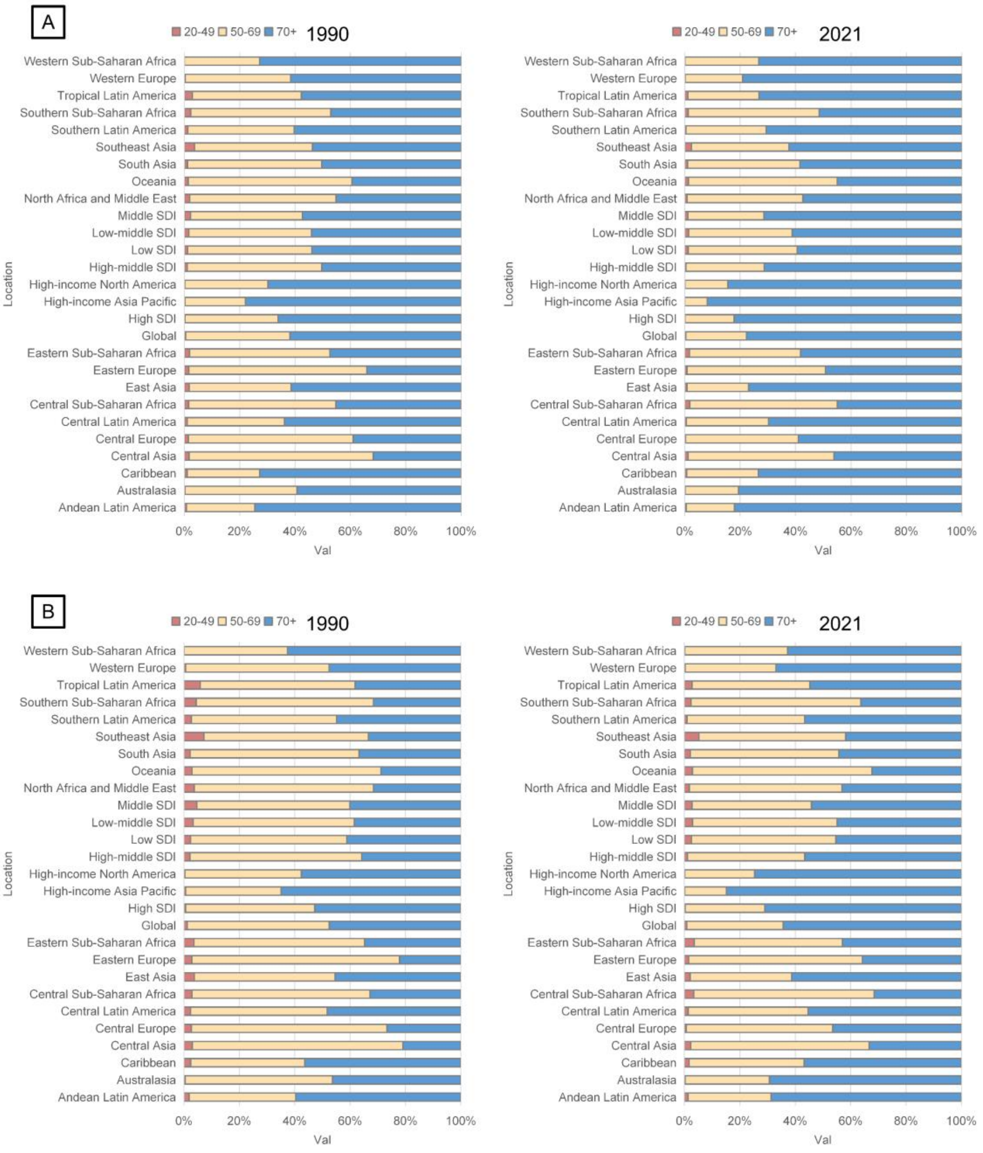

Supplementary Fig. 7. Proportion of Age Subgroups of Lung Cancer Deaths (A) and DALYs (B) Attributable to Occupational Asbestos Exposure by Region in 1990 and 2021.

DALY, disability-adjusted life years.

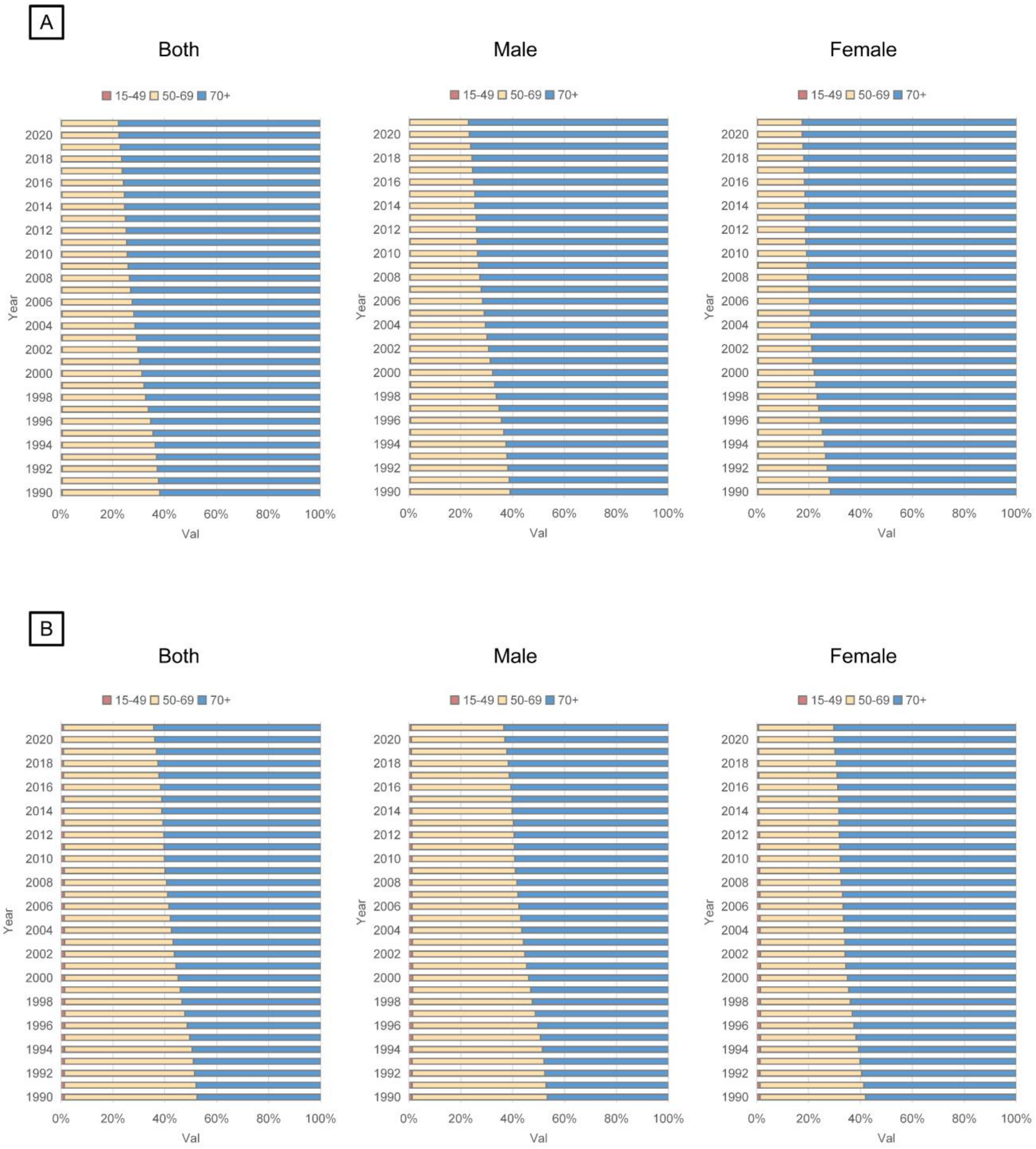

Supplementary Fig. 8. Proportion of Age Subgroups of Lung Cancer Deaths (A) and DALYs (B) Attributable to Occupational Asbestos Exposure at Global Level by Sex and Year.

DALY, disability-adjusted life years.

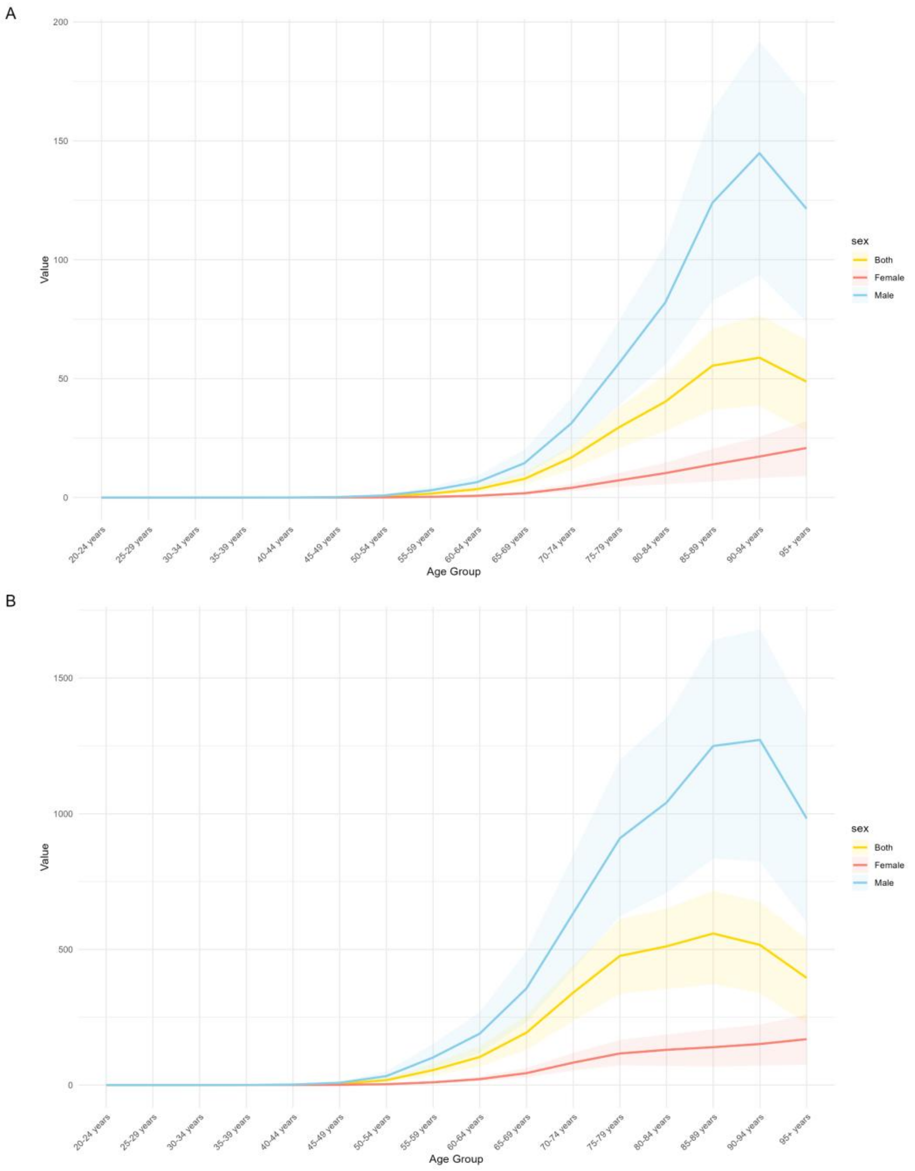

Supplementary Fig. 9. Age-Specific Rates of Global Deaths (A) and DALYs (B) of Lung Cancer Attributable to Occupational Asbestos Exposure with 95% UI by Sex in 2021.

DALYs, disability-adjusted life-years; UI, uncertainty interval.

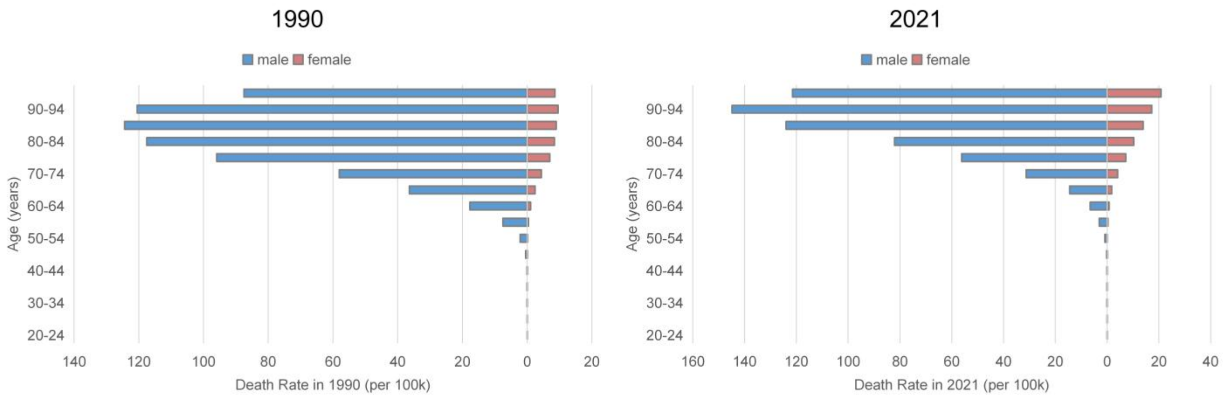

Supplementary Fig. 10. Comparison of Age-Specific Rates of Death Between Female and Male Patients in 1990 and 2021.

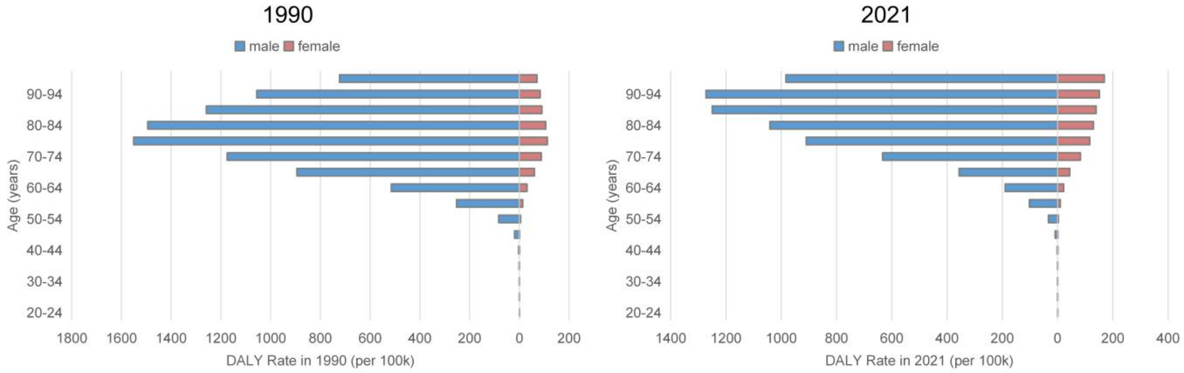

Supplementary Fig. 11. Comparison of Age-Specific Rates of DALY Between Female and Male Patients in 1990 and 2021.

DALY, disability-adjusted life year
